# MYC regulates fatty acid metabolism through a multigenic program in claudin-low triple negative breast cancer

**DOI:** 10.1038/s41416-019-0711-3

**Published:** 2020-01-16

**Authors:** Jessica C. Casciano, Caroline Perry, Adam J. Cohen-Nowak, Katelyn D. Miller, Johan Vande Voorde, Qifeng Zhang, Susan Chalmers, Mairi E. Sandison, Qin Liu, Ann Hedley, Tony McBryan, Hsin-Yao Tang, Nicole Gorman, Thomas Beer, David W. Speicher, Peter D. Adams, Xuefeng Liu, Richard Schlegel, John G. McCarron, Michael J. O. Wakelam, Eyal Gottlieb, Andrew V. Kossenkov, Zachary T. Schug

**Affiliations:** 1 The Wistar Institute, Molecular and Cellular Oncogenesis, 3601 Spruce Street, Philadelphia, PA 19104 USA; 2 Perelman School of Medicine, University of Pennsylvania, Philadelphia, PA 19104 USA; 3 The Beatson Institute, Garscube Estate, Switchback Road, Glasgow, G61 1BD UK; 4 The Babraham Institute, Babraham Research Campus, Cambridge, CB22 3AT UK; 5 Strathclyde Institute of Pharmacy and Biomedical Sciences, University of Strathclyde, SIPBS Building, 161 Cathedral Street, Glasgow, G4 0RE UK; 6 Department of Biomedical Engineering, University of Strathclyde, Wolfson Centre, 106 Rottenrow, Glasgow, G4 0NW UK; 7 Institute of Cancer Sciences, College of Medical, Veterinary, and Life Sciences, University of Glasgow, Glasgow, G61 1BD UK; 8 Sanford Burnham Prebys Medical Discovery Institute, 10901 North Torrey Pines Road, La Jolla, CA 92037 USA; 9 Center for Cell Reprogramming, Lombardi Comprehensive Cancer Center, Georgetown University Medical Center, 3900 Reservoir Road, Washington D.C., 20057 USA; 10 The Ruth and Bruce Rappaport Faculty of Medicine, Technion - Israel Institute of Technology, 1 Efron St. Bat Galim, 3525433 Haifa, Israel

**Keywords:** Cancer metabolism, Breast cancer, Oncogenes, Epithelial-mesenchymal transition, Metabolomics

## Abstract

**Background:**

Recent studies have suggested that fatty acid oxidation (FAO) is a key metabolic pathway for the growth of triple negative breast cancers (TNBCs), particularly those that have high expression of MYC. However, the underlying mechanism by which MYC promotes FAO remains poorly understood.

**Methods:**

We used a combination of metabolomics, transcriptomics, bioinformatics, and microscopy to elucidate a potential mechanism by which MYC regulates FAO in TNBC.

**Results:**

We propose that MYC induces a multigenic program that involves changes in intracellular calcium signalling and fatty acid metabolism. We determined key roles for fatty acid transporters (CD36), lipases (LPL), and kinases (PDGFRB, CAMKK2, and AMPK) that each contribute to promoting FAO in human mammary epithelial cells that express oncogenic levels of MYC. Bioinformatic analysis further showed that this multigenic program is highly expressed and predicts poor survival in the claudin-low molecular subtype of TNBC, but not other subtypes of TNBCs, suggesting that efforts to target FAO in the clinic may best serve claudin-low TNBC patients.

**Conclusion:**

We identified critical pieces of the FAO machinery that have the potential to be targeted for improved treatment of patients with TNBC, especially the claudin-low molecular subtype.

## Background

A recent study analysed the copy number and gene expression changes in over 2000 breast tumours.^1,2^ One of the most commonly and highly amplified genes in breast cancer is MYC.^1,3^ MYC is a transcription factor that activates genes involved in cell cycle regulation, cell growth, protein synthesis, mitochondrial function, and metabolism. MYC amplification occurs in ~25% of all breast cancers and occurs more frequently in triple negative breast cancer (TNBC) (up to 50%).^4,5^ MYC gene amplification is associated with risk of relapse, poor prognosis, and death.^6,7^

MYC is a known regulator of metabolic reprogramming in cancer.^8,9^ A recent study reported that TNBC cells with high expression of MYC have high rates of fatty acid β-oxidation (FAO).^10^ They further demonstrated that pharmacological inhibition of FAO with etomoxir impairs the growth of TNBC patient-derived xenografts that have high MYC expression but not those with low MYC expression, suggesting that targeting FAO may be a viable treatment option for TNBC patients with high expression of MYC.^10^ Unfortunately, the safety of etomoxir has been called into question during clinical trials due to reports of liver toxicity and a recent study showed that etomoxir can cause severe oxidative stress in an off-target (non-CPT1A) dependent manner.^11,12^ There is therefore unmet need for better, safer inhibitors of FAO. Other targets associated with FAO have emerged more recently. For instance, FAO has been shown to promote SRC activation and metastasis in TNBC,^13^ while a separate study showed that CUB-domain containing protein 1 (CDCP1) inhibits acyl-CoA synthetases which then stimulates FAO in TNBC cell lines.^14^ However, the role of CDCP1 in breast is controversial with conflicting reports.^15,16^

The goal of our study was not to further prove the importance of FAO in TNBC, but to elucidate the mechanistic link between MYC and the activation of FAO and to identify new actionable targets for blocking FAO in TNBC. We used human mammary epithelial (HME) cells that express oncogenic levels of MYC as a model system to cleanly delineate how MYC leads to enhanced FAO. Our results indicate that MYC alters calcium (Ca^2+^) signalling that then promotes FAO by activating a Ca^2+^-CAMKK2-AMPK signalling axis. We also identified an important role for fatty acid transporter CD36, which mediates fatty acid uptake in TNBC. MYC HME cells exhibited a 10-fold increase in cell migration compared to isogenic cell lines expressing telomerase reverse transcriptase (TERT) or HER2. The migration of MYC HME cells could be inhibited by targeting CD36, CAMKK2, or FAO suggesting that uptake and oxidation of fatty acids may be an important metabolic pathway for supporting the energy demanding process of cell migration. We find that CD36 and other fatty acid metabolism genes (i.e. LPL, PDK4, FABP4) are highly co-expressed in TNBC, particularly in the claudin-low subtype of TNBC where their high expression predicts poor survival. Collectively, our studies helped to identify targets that could be considered as new options for blocking FAO in TNBC and suggests that targeting FAO may be most beneficial for claudin-low TNBC patients.

## Methods

### Cell culture, siRNA transfection, and lentiviral transduction

HME cells were cultured in 1× HMEC Basal Serum-Free Medium (Thermo Fisher Scientific) supplemented with the HMEC Supplement Kit (Thermo Fisher Scientific) and 1× penicillin–streptomycin (Corning). MDA-MB-468 (ATCC), T47D (ATCC), BT474 (ATCC), MDA-MB-231 (ATCC), Cal120 (DSMZ), Cal51 (DSMZ), BT549 (ATCC) cells were cultured in 1× DMEM/F-12 50/50 supplemented with 10% foetal bovine serum (FBS) (Life Technologies) and 1× penicillin–streptomycin. Hs578t (ATCC) cells were cultured in 1× DMEM/F-12 50/50 supplemented with 0.01 mg/ml bovine insulin (Sigma), 10% FBS, and 1× penicillin–streptomycin. SUM159PT (Asterand Bioscience) cells were cultured in 1× DMEM/F-12 50/50 supplemented with 5% FBS, hydrocortisone (1 μg/ml), and human insulin (5 μg/ml). siRNA transfections were performed by reverse transfection using RNAiMAX. All siRNAs were obtained from Qiagen and used as pools of four individual siRNAs. MYC+pTRIPZ, MYC+shCD36#2, and MYC+shCD36#3 were generated using lentivirus produced by HEK293T cells transfected (Lipofectamine 2000) with psPAX2, pVSV-G, and the corresponding pTRIPZ vector (GE Healthcare). MYC HME cell pools were selected using puromycin. Doxycycline (0.5 μg/ml) was used to induce transcription of shRNAs.

### Western blotting

Cells were lysed in 1× Bolt LDS Sample Buffer (Life Technologies) supplemented with 50 mM dithiothreitol (DTT). Lysates were heated at 95 °C for 5 min and then centrifuged at 10,000×*g* for 10 min. Lysates were then resolved using Bolt 4–12% Bis-Tris Plus precast polyacrylamide gels (Life Technologies) for 30 min at 200 V and blotted onto nitrocellulose membranes for 1 h at 10 V using the Mini Blot Module transfer system (Life Technologies). The blots were then blocked using 5% milk in Tris buffered saline solution with tween (TBST) for 1 h at room temperature. Blots were incubated with primary antibodies overnight at 4 °C. Primary antibodies were used at a 1:1000 dilution in 1% bovine serum albumin (BSA) and 0.05% sodium azide in TBST. Antibodies were purchased from the following vendors: Actin (Abcam #8226), TERT, HER2 (Cell Signaling #4290), MYC (Cell Signaling #5605), tubulin (Sigma HPA043640), ER (Cell Signaling #8644), PR (Cell Signaling #8757), EGFR (Cell Signaling #4267), AMPK (Cell Signaling #2532), P-AMPK (Cell Signaling #2535), P-ACC (Cell Signaling #3661), CAMKK2 (Santa Cruz #100364 and Abnova #H00010645), CDH1 (Cell Signaling #5296), and PDGFRB (Cell Signaling #3169). Secondary antibodies were purchased from Li-Cor Biosciences (goat anti-mouse #926-32210 and donkey anti-rabbit #926-68073) and diluted to a 1:10,000 solution in TBST. Incubation with the secondary antibody occurred at room temperature for 1 h. Blots were imaged using a Li-Cor Odyssey infrared imager.

### Quantitative PCR (qRT-PCR)

Total RNA was isolated using the RNeasy Mini Kit (Qiagen) and reverse transcribed using the SuperScript IV VILO Master Mix (Life Technologies). cDNA was amplified via the Fast SYBR Green Master Mix (Life Technologies) using the ABI 7500 Fast qPCR system (Thermo Fisher Scientific). Results were analysed using the ABI 7500 software v2.0.6. Relative expression levels of target genes were determined by normalisation to the β-actin gene using the ΔΔC_t_ method.

For quantification of mitochondrial DNA, mtDNA was isolated from HME cells using the Mitochondrial DNA Isolation Kit (Abcam; ab65321). Genomic DNA (gDNA) was isolated from HME cells using a gDNA purification kit (Thermo Scientific). qPCR was performed using the ABI 7500 Fast qPCR system (Thermo Fisher Scientific) and results were analysed using the ABI 7500 software v2.0.6. Relative expression levels of the mitochondrial genes tRNA^Leu(UUR)^ and 16S rRNA were determined by normalisation to the nuclear gene β2-microglobulin using the ΔΔC_t_ method as previously described.^17,18^

### Flow cytometry

For MitoTracker Green staining HME cells were pelleted, washed with ice-cold PBS, and resuspended in 1× HME cells Basal Serum-Free Medium (Thermo Fisher Scientific) and incubated with 20 nM MitoTracker Green FM (Thermo Fisher Scientific). Cells were then stained with PI (Alfa Aesar). Cells were sorted on a FACSCalibur (Becton-Dickinson) flow cytometer using CellQuest software. Cells were first sorted for PI staining; PI-positive cells were excluded from analysis. Cells were then sorted for MitoTracker Green staining. The geometric mean of MitoTracker Green intensity was used for analysis. Figure presentation was completed using FlowJo software.

For cell death/cell cycle analysis via PI staining, HME cells were treated with 10 µM STO-609 or 150 µM Etomoxir for 48 h. Cells and cell medium were pelleted, washed with ice-cold PBS and then fixed with ice-cold 70% ethanol. Cells were washed once more with ice-cold PBS prior to RNA digestion. Cells were then stained with PI. Cells were sorted on a FACSCalibur (Becton-Dickinson) flow cytometer using CellQuest software. Cells were first sorted for PI staining and a cell cycle profile was created based on PI fluorescence intensity. The percentage of cells in each segment of the cell cycle profile (Sub-G1, G0/G1, S, or G2/M) was used for analysis. Data analysis and figure presentation was completed using FlowJo software.

For cell surface receptor expression analysis, cells were detached using cell dissociation buffer (Thermo Fisher Scientific) and washed in ice-cold PBS. Cells were washed in stain buffer (PBS and 5% BSA). Cells were incubated for 30 min on ice in primary antibody CD36 (Epitomics #S1249; now discontinued) or GFP (Clontech) and then washed in stain buffer. For CD36 and GFP staining, Alexa Fluor 488 was added for 15 min on ice before washing the cells in stain buffer. Cells were analysed on a FACSCalibur (Becton-Dickinson) flow cytometer using CellQuest software.

### Confocal microscopy

MYC and TERT HME cells were grown in 6-well plates on glass coverslips. Cells were washed with PBS and fixed in 4% paraformaldehyde at room temperature. Coverslips were washed in PBS and blocked with 10% FBS and incubated overnight with anti-human CD36 (Epitomics #S1249; now discontinued) in 5% BSA. Coverslips were incubated with Alexa Fluor 488 anti-mouse IgG (Molecular Probes) in 5% BSA, washed in PBS and mounted in immunofluorescence mounting medium containing DAPI (Vectashield; Vector Laboratories, Servion, Switzerland). Specimens were analysed using a confocal microscope (Leica SP2 (DMIRBE) laser scanning confocal with Leica SP2 software) equipped with a 63×/1.32.ph3 oil HCX PL APO lens (Leica, Milton Keynes, UK).

### Time-lapse imaging of fatty acid uptake

HME cells were plated on thin layer matrigel-coated glass 6-well plates and treated with 0.5× Loading Buffer containing BODIPY-labelled dodecanoic acid. Loading Buffer was prepared according to the manufacturer’s instructions for the QBT Fatty Acid Uptake Assay Kit (Molecular Devices). Briefly, the contents of one vial containing BODIPY-labelled dodecanoic acid was dissolved in 10 ml of 1× HMEC Basal Serum-Free Medium (Thermo Fisher Scientific) + 0.2% fatty acid-free BSA (Sigma) to create 1× Loading Buffer. HME cells were imaged for 6 h using a Nikon TE300 Automated Inverted Microscope equipped with a 40× objective and a Hamamatsu ORCA camera. Images were captured every 5 min with a 400-ms exposure. For time-lapse imaging of STO-609- (10 μM) and etomoxir (40 μM)-treated cells, drugs were added simultaneously with 0.5× Loading Buffer. Fluorescence intensity and cell migration was tracked and calculated for individual cells using the Nikon NIS Elements software, version 4.2.

### Carbon-13 stable isotope tracing experiments and metabolomics

All metabolomic and lipidomic studies were performed as previously described with minor differences.^19,20^ All metabolomic experiments were performed in serum-like modified Eagle’s medium (SMEM), the formula of which has been previously described.^19,21^ SMEM contains 54 different nutrients that are found in the bloodstream at concentrations that are physiologically relevant to humans. SMEM was supplemented with the HMEC Supplement Kit from Invitrogen (Life Technologies). Cells were incubated in uniformly labelled ^13^C-palmitate (0.05 mM), ^13^C-glucose (5.5 mM), or ^13^C-glutamine (0.65 mM) for 30 min, 4 h, or 8 h as indicated in the figure legends. For intracellular extracts, after incubation, the SMEM medium was aspirated and cells were washed once in ice-cold PBS. Metabolites were extracted by adding a solution of methanol/acetonitrile/water (5:3:2) to the well. Plates were incubated at 4 °C for 5 min on a rocker and then the extraction solution was collected. The metabolite extract was cleared by centrifuging at 15,000×*g* for 10 min at 4 °C. Supernatants were transferred to LC-MS silanised glass vials with PTFE caps and either run immediately on the LC-MS or stored at −80 °C.

For analysis of nutrient uptake and efflux, the medium was collected after 24 h and diluted 50-fold into extraction solution (described above). Extracted medium was vortexed on a thermomixer for 10 min at 4 °C before being frozen at −80 °C overnight. The extracted medium was thawed on ice the next day and following centrifugation, the cleared supernatants were transferred to silanised glass vials and either run immediately by LC-MS or stored at −80 °C.

LC-MS analysis was performed on a Q Exactive Hybrid Quadrupole-Orbitrap HF-X MS (Thermo Fisher Scientific) equipped with a HESI II probe and coupled to a Vanquish Horizon UHPLC system (Thermo Fisher Scientific). A total of 0.002 ml of sample is injected and separated by HILIC chromatography on a ZIC-pHILIC 2.1-mm. Samples are separated by ammonium carbonate, 0.1% ammonium hydroxide, pH 9.2, and mobile phase B is acetonitrile. The LC was run at a flow rate of 0.2 ml/min and the gradient used was as follows: 0 min, 85% B; 2 min, 85% B; 17 min, 20% B; 17.1 min, 85% B; and 26 min, 85% B. The column was maintained at 45 °C and the mobile phase was also pre-heated at 45 °C before flowing into the column. The relevant MS parameters were as listed: sheath gas, 40; auxiliary gas, 10; sweep gas, 1; auxiliary gas heater temperature, 350 °C; spray voltage, 3.5 kV for the positive mode and 3.2 kV for the negative mode. Capillary temperature was set at 325 °C, and funnel RF level at 40. Samples were analysed in full MS scan with polarity switching at scan range 65–975 *m*/*z*; 120,000 resolution; automated gain control (AGC) target of 1E6; and maximum injection time (max IT) of 100 ms. Identification and quantitation of metabolites was performed using an annotated compound library and TraceFinder 4.1 software. The “M+X” nomenclature refers to the isotopolog for that given metabolite. Isotopologs are chemically identical metabolites that differ only in their number of carbon-13 atoms. For instance, “M+2 citrate” means that two of the six carbons in citrate are carbon-13 while the other four are carbon-12. “M+4 citrate” means that four of the six carbons in citrate are carbon-13 while the other two are carbon-12.

### Lipidomics

Cell pellets were washed twice with cold degassed PBS and resuspended in methanol before transfer to silanised glass tubes. Samples were spiked with 400 ng 12:0/12:0-PC, 100 ng 17:0-LPC, 300 ng 12:0/12:0-PE, and 100 ng 17:1-LPE; 0.88% NaCl; and chloroform. The mixture was vortexed for 20 s at room temperature and sonicated in an ice-cold water bath for 2 min. Samples were centrifuged at 1100 rpm at 4 °C for 15 min. The lower phase was collected. The upper phase was extracted with synthetic lower phase (mix chloroform/methanol/0.88% NaCl at a volume ratio of 2:1:1, after phase separation, take the lower phase as synthetic lower phase for second extraction of lipid). The resulting lower phase was combined and dried under vacuum at room temperature with SpeedVac (Thermo Scientific) and re-dissolved in chloroform. The final product was injected for LC-MS/MS analysis using a Thermo Orbitrap Elite system (Thermo Fisher Scientific) hyphenated with a five-channel online degasser, four-pump, column oven, and autosampler with cooler Prominence HPLC system (Shimadzu) for lipids analysis. In detail, lipid classes were separated on a normal-phase silica gel column (2.1 × 150 mm, 4micro, MicoSolv Technology) with hexane/dichloromethane/chloroform/methanol/acetonitrile/water/ethylamine solvent gradient based on the polarity of head group. High resolution (240k at *m*/*z* 400)/accurate mass (with mass accuracy < 5 ppm) and tandem MS (collision-induced fragmentation) were used for molecular species identification and quantification. The identity of lipid was further confirmed by reference to appropriate lipids standards.

### Oxygen consumption rate and extracellular acidification rate measurements

Cells were seeded for 24 h prior to the assay and were 90–100% confluent. For FAO measurements, cells were washed twice in KHB buffer (111 mM NaCl, 4.7 mM KCl, 2 mM MgSO_4_, 1.25 mM CaCl_2_, 1.2 mM Na_2_HPO_4_, 100 μM l-carnitine, 5 mM HEPES and pH adjusted to 7.4 with NaOH) before the addition of 100 μM BSA-conjugated palmitate. Cells were incubated for 1  h to adjust to the buffer before analysis using a Seahorse Bioanalyzer XF24 (Agilent). Antimycin A and rotenone were added to establish the background oxygen consumption rate (OCR) and subtracted from the basal OCR that is reported. For platelet-derived growth factor receptor beta (PDGFRB) and epidermal growth factor receptor (EGFR) studies on FAO, cells were pre-treated for 1 h with 25 ng/ml PDGF-BB or EGF before addition of 100 μM α-cyclodextrin conjugated palmitate. STO-609 was added at 10 μM and etomoxir at 40 μM.

For extracellular acidification rate (ECAR) measurements, TERT and MYC HME cells were seeded in HME serum-free medium in a 96-well Seahorse Bioanalyzer plate 24 h prior to analysis. Cells were washed once with PBS before addition of Seahorse medium supplemented with 5.50 mM glucose, 0.65 mM glutamine, and 0.10 mM pyruvate. ECAR was measured using a XFe96 Seahorse Bioanalyzer. Data were analysed using the Seahorse Wave 2.6 software.

### Lipoprotein lipase activity assay

Lipoprotein lipase (LPL) activity assay was performed according to manufacturer’s protocol (Sigma). Briefly, a master reaction mix was prepared that contained assay buffer and an LPL substrate emulsion and 195 μl was pipetted into a 96-well black clear bottom (flat) plate. A total of 5 μl of HME cell lysate was added using a multichannel pipette and the plate was immediately placed on a fluorimeter and read (Ex = 365; Em = 485) every 80 s for 1 h at 37 °C. Cell lysate-free wells were used for background subtraction. A Lowry assay was used to assess protein concentration in the cell lysates.

### Carbon-14 FAO assay

HME cells were seeded for 24 h prior to the FAO assay. The cells were washed in PBS and fresh medium supplemented with 200 μM carnitine and 1.2 μCi (20 μM) U-^14^C-palmitate conjugated with α-cyclodextrin and incubated for 4 h at 37 °C. Whatman paper soaked in 5 N NaOH was attached to the lid of each dish and 2.6 N HClO_3_ was added to the medium to evolve and capture the ^14^CO_2_ over 1 h. Whatman paper was counted directly in scintillation fluid. For acid soluble metabolites (ASMs), the medium was removed and 4 N KOH was added to hydrolyse acyl-CoA esters (saponify) at 60 °C for 30 min; 1 M NaC_2_H_3_O_2_ and 3 N H_2_SO_4_ were added and samples were vortexed and transferred to a glass vial. A 2:1 mixture of CHCl_3_:CH_3_OH was added and samples were vortexed and then 4 mM MgCl_2_ was added and samples were vortexed again before a short spin to separate the phases. The upper (aqueous) phase was added to scintillation fluid and using a scintillation counter. Lowry assay was used to assay protein content and normalise the data.

### RNA-sequencing and ingenuity pathway analysis

RNA was isolated using a Qiagen RNeasy kit. Ribodepletion was performed using an Epicentre Ribo Zero kit. Libraries were prepared according to the Illumina TruSeq protocol and sequenced on an Illumina GAIIx. Tophat 1.4.1 align versus hg18 using ensembl (ncbi36.54 transcriptome). Cuffdiff 2.0.2 was used to do pairwise comparisons with fragment bias and multi-read corrections. Pairwise sample comparisons were performed using Cuffdiff and False Discovery Rate (FDR) estimated by Benjamini–Hochberg method was used to create a set of significant genes that pass FDR <5% cut-off. All the genes that met the FDR 5% cut-off also had at least 4.8-fold change difference in all comparisons. Samples were normalised using upper quartile normalisation and considered significant at FDR < 5%. Gene networks, canonical pathways, and functional analyses were generated through the use of ingenuity pathway analysis (IPA) (https://www.qiagenbioinformatics.com/products/ingenuity-pathway-analysis).^22^ There was only one replicate per condition, however, since Cuffdiff uses beta negative binomial distribution as an underlying statistical model, the significance takes into account the read count nature of RNA sequencing data and gives an extra level of confidence compared to using only fold change.

### Data mining and human patient data

All patient tumour mRNA expression data, *z*-scores, copy number gains, and gene amplification data were obtained from www.cbioportal.org.^23,24^ Breast cancer patient tumours were classified based on the molecular subtypes as called by the METABRIC database and PAM50+claudin-low.^1^ All METABRIC expression data and patient metadata are freely available through www.cbioportal.org.

### Calcium imaging

Cells were loaded (20 min, 37 °C) with fluo4 AM (10 µM; Life Technologies, Paisley, UK) and membrane permeant caged IP3 (10 µM; SiChem) in a solution containing (mM) sodium glutamate, 80; NaCl, 54; KCl, 5; MgCl_2_, 1; CaCl_2_, 0.1, Hepes, 10; glucose, 10; and 0.2, EDTA (to remove heavy metals); the pH adjusted at room temperature to 7.3 using NaOH.

After loading, cells were washed and imaged in medium (HME cells Basal Serum-Free Medium supplemented with the HMEC Supplement Kit and 1× penicillin–streptomycin).

Two-dimensional [Ca^2+^]_c_ images were obtained using a wide-field digital imaging system. Excitation illumination was provided by a monochomator (PTI Inc., West Sussex, UK) to give 490 nm (bandpass 5 nm), coupled via a liquid light guide to a Nikon TE2000U microscope (Nikon UK, Surrey England). Fluorescence emission was collected by the objective lens (×40, NA 1.3) and transmitted to a cooled, back-illuminated, frame transfer CCD camera with on-chip electron multiplication (Cascade 512B; Photometrics Tuscan, AZ, USA) controlled by Winfluor (University of Strathclyde, Glasgow, UK). Images were collected at a frequency of 10 Hz unless otherwise indicated. Photolysis of caged-IP_3_ was achieved using a frequency-tripled ND:Yag (wavelength 355 nm) laser attached directly to the microscope (Rapp Optoelektronic, Hamburg, Germany). The position of the photolysis site (~2 µm diameter) was computer controlled (Rapp Optoelektronic). The duration of the photolysis pulse was 1 ms and energy, measured at the objective, 100 µW.

[Ca^2+^] images were analysed using the program Metamorph 7.5 (Molecular Devices Ltd., Wokingham, U.K.) or Image Pro 7.0 (Media Cybernetics, MD). To compensate for variations in fluorescence across the imaging field, e.g., from irregularities in focus of the cells, fluorescence signals were background subtracted and expressed as ratios (*F*/*F*0) of fluorescence counts (*F*) relative to baseline (control) values (taken as 1) before stimulation (*F*0).

PDGF-BB was diluted in 1% BSA–PBS and an aliquot injected (1:100 dilution) into the imaging bath to give 100 ng/ml. Responders were classified as those cells with a peak *F*/*F*_0_ of >1.15 that occurred shortly after PDGF exposure (within 30 s).

## Results

### Generation of HME cells expressing TERT, MYC, or HER2

**Fig. 1:**
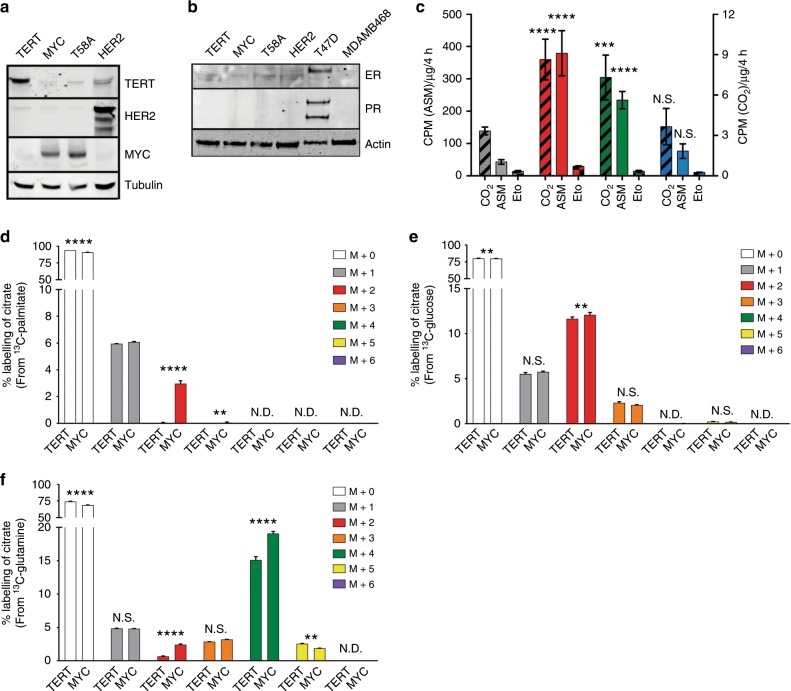
MYC expression is sufficient to induce fatty acid oxidation in human mammary epithelial cells. **a** Immunoblot analysis of TERT, HER2, and MYC protein expression in HME cell lines (stable pools). **b** Immunoblot analysis of oestrogen receptor (ER) and progesterone receptor (PR) in HME cells and human breast cancer cell lines. T47D breast cancer cells are positive for both ER and PR and served as a control for hormone receptor-positive cells. MDA-MB-468 lack expression of ER, PR, and HER2 and served as a control for triple negative breast cancer cells. **c** FAO of U-^14^C-palmitate in HME cells. Left *y*-axis represents carbon-14 labelling of acid soluble metabolites (ASM; solid bars). Right *y*-axis represents the capture of ^14^CO_2_ (black slashed bars) from HME cells, a readout of FAO. Etomoxir treatment is represented by the right *y*-axis and shows the inhibition of ^14^CO_2_ production from U-^14^C-palmitate (checkered bars). Throughout the manuscript, TERT = grey, MYC = red, T58A = green, HER2 = blue; *n* ≥ 3 independent experiments. Error bars represent mean ± standard deviation (s.d.). *p* Values were calculated using an unpaired Student’s *t*-test and are relative to TERT HME cells for each respective measurement. **d–f** LC-MS-based quantification of citrate labelling from cells treated with 0.05 mM U-^13^C-palmitate (**d**), 5.5 mM U-^13^C-glucose (**e**), and 0.65 mM U-^13^C-glutamine (**f**) for 30 min. The carbon-13 labelling is presented as a percent abundance of each isotopolog relative to the total citrate pool; *n* = 3 independent samples. Samples were measured in a single run on the mass spectrometer. Adjusted *p* values were calculated using an ordinary two-way ANOVA and Sidak’s multiple comparison test for each isotopolog; *p < 0.05, **p < 0.01, ***p < 0.001, ****p < 0.0001, N.S. = not significant, N.D. = not detected.

HME cells have been routinely used as a model system to study the oncogenic transformation of non-immortalised breast epithelial cells to immortalised transformed cancer cells.^25–27^ Furthermore, HME cells lack expression of the oestrogen receptor (ER) and therefore provides the opportunity to model the development of ER-negative breast cancers.^28^ A more recent study showed that the phenotype and cellular behaviour that emerged in a separate model of MYC expressing HME cells was very similar to that of MDA-MB-231 cells, a TNBC cell line.^29^ To identify novel targets associated with the activation of FAO by MYC, we used post-selection HME cells that stably express wild-type *MYC* (MYC HME cells) or a mutant version of *MYC*^T58A^ (T58A HME cells) that has a slightly longer half-life (Fig. 1a).^30,31^ MYC is able to immortalise HME cells in part through the stabilisation of TERT.^25^ To ensure that the effects we were observing in the two MYC HME cells lines were specific to MYC expression and not due to TERT, we also used an isogenic HME cell line that instead had been immortalised by direct stable expression of *TERT* (TERT HME cells; Fig. 1a).^31,32^ Lastly, we introduced human epidermal growth factor receptor 2 (*HER2*) into TERT HME cells to produce HER2-overexpressing HME cells (HER2 HME cells). HER2 is an oncogene that is commonly amplified in ~20% of breast cancers, is mutually exclusive with TNBC, and serves as a separate model of oncogene overexpression that is relevant to breast cancer (Fig. 1a). All four pools of isogenic HME cells cell lines (TERT, MYC, T58A, and HER2) are negative for ER and progesterone receptor (PR) (Fig. 1b). We therefore used MYC HME cells and T58A HME cells as representative models of TNBC in which we could cleanly delineate the effects of MYC on FAO.

### MYC is sufficient to stimulate FAO in HME cells

We initially wanted to test if MYC is sufficient to induce FAO in HME cells. To measure FAO, we incubated HME cells in uniformly labelled carbon-14 palmitate (^14^C_16_-palmitate). We then measured ^14^CO_2_ production by the HME cells as well as intracellular levels of ^14^C-labelled ASMs (Fig. 1c). ASMs are defined as carbon-14-labelled intracellular polar metabolites that are formed from the catabolism of ^14^C_16_-palmitate, such as acetyl-CoA and tricarboxylic acid (TCA) cycle intermediates. Importantly, all FAO activity was blocked by the addition of etomoxir, an inhibitor of the rate limiting enzyme of FAO, carnitine palmitoyltransferase 1A (CPT1A) (Fig. 1c). This FAO assay showed that MYC and T58A HME cells have significantly higher rates of FAO compared to TERT and HER2 HME cells. These results were also confirmed by measuring OCRs on a Seahorse Bioanalyzer when palmitate conjugates were provided as a sole fuel substrate (Supplementary Fig. S1a).

Although FAO assays showed that MYC and T58A HME cells have a higher capacity to oxidise fatty acids, it did not inform about the contribution of fatty acids to mitochondrial metabolism compared to other fuel sources such as glucose or glutamine. To address this, we incubated cells for a short period (30 min) in uniformly labelled carbon-13 (^13^C) glucose, glutamine, and palmitate to compare the oxidation of these three nutrient sources using citrate labelling as a readout. While we did detect ^13^C-labelling of citrate by ^13^C-palmitate in MYC HME cells, we barely detected labelling of citrate by ^13^C-palmitate in TERT HME cells, which agrees with the results in Fig. 1c and Supplementary Fig. S1a, and further suggests that TERT HME cells engage very low levels of FAO (Fig. 1d and Supplementary Fig. S1b). Although statistically significant, we detected only subtle differences in citrate labelling from ^13^C-glucose (Fig. 1e and Supplementary Fig. S1c). ^13^C-palmitate was oxidised at ~25% the rate of ^13^C-glucose; however, this is likely an underestimation of actual level of FAO in MYC HME cells, since there are other fatty acids present in the culture medium which were also likely oxidised along with the 50 μM ^13^C-palmitate that was provided. We repeated our metabolic flux analysis by measuring citrate labelling for longer periods (4 and 8 h) after ^13^C-palmitate addition in TERT, MYC, and HER2 HME cells. By 8 h, >22% of the citrate pool was labelled by ^13^C-palmitate in MYC HME cells, while <6% was labelled in either TERT or HER2 HME cells (Supplementary Fig. S1d). Similar labelling patterns were measured for other TCA cycle intermediates, including α-ketoglutarate and malate (Supplementary Fig. S1e, f). These results suggest that MYC expression was sufficient to induce FAO in human breast cells.

One of the more striking findings from the ^13^C tracer studies was that MYC HME cells displayed significantly higher labelling of citrate by ^13^C-labelled glutamine, suggesting high flux of glutamine carbon into the TCA cycle (Fig. 1f and Supplementary Fig. S1g). It has been well documented that MYC induces upregulation of glutaminase expression^33^ and we find that mitochondrial-localised glutaminase 2 (*GLS2*) expression was upregulated in MYC and T58A HME cells in a MYC-dependent manner (Supplementary Fig. S1h). Interestingly, glutamine uptake was not different between TERT and MYC HME cells; however, glutamate efflux was substantially lower in MYC HME cells (Supplementary Fig. S1i, j). Lower glutamate efflux would be compatible with our observations of higher GLS2 expression and higher flux of glutamine carbon into the TCA cycle. Altogether, these findings could suggest that TERT HME cells are more dependent on an alternative metabolic pathway for ATP production and indeed we find that TERT HME cells consume slightly more glucose and secrete higher amounts of lactate suggesting that pyruvate may be diverted to lactate instead of to the mitochondria in TERT HME cells (Supplementary Fig. S1k, l). We also used a Seahorse bioanalyzer to measure the ECAR as an alternative method to investigate lactate efflux from TERT and MYC HME cells (Supplementary Fig. S1m). This separate approach showed that TERT HME cells had a slightly higher ECAR compared to MYC HME cells. Importantly, the higher flux of palmitate and glutamine into the TCA cycle of MYC HME cells does not appear to be due to the presence of more mitochondria in MYC HME cells since there are no differences in Mitotracker Green labelling or mitochondrial DNA content between the four HME cell lines (Supplementary Fig. S1n–p).

### MYC HME cells are enriched with fatty acid metabolism genes and have phospholipid profiles indicative of fatty acid uptake

**Fig. 2:**
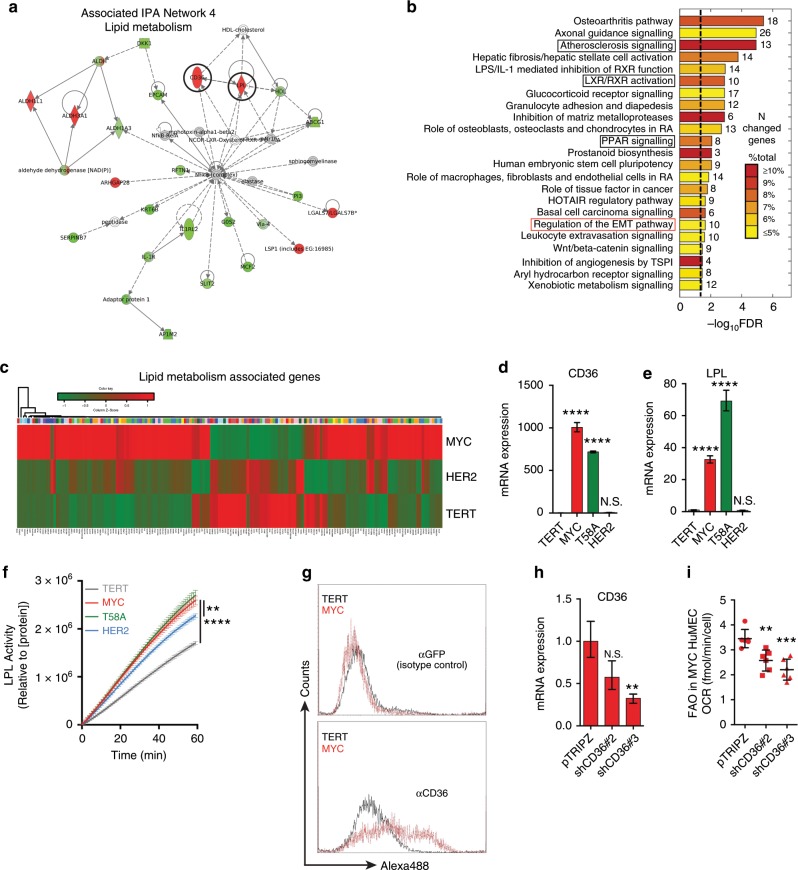
MYC HME cells use LPL and CD36 to mobilise and take up fatty acids. **a** Interaction map of the lipid metabolism gene network in MYC HME cells. Ingenuity Pathway Analysis was used to create the interaction map. Black circles highlight lipid metabolism genes CD36 and LPL. **b** Top canonical signalling pathways in MYC versus TERT HME cells based on RNA sequencing data (377 genes; FDR < 5%; minimum fold change: 6.81). Ingenuity Pathway Analysis was used to identify the pathways. Black boxes highlight gene ontologies associated with lipid metabolism. The value next to each bar represents the number of altered genes within the indicated signalling pathway. The colour of the bar indicates the percent of genes within the pathway that are differentially regulated. The black dotted line marks significance (FDR < 5%). **c** Heat map showing the relative expression of 163 genes associated with lipid metabolism in TERT, MYC, and HER2 HME cells (1.4 over all upregulated genes in MYC HME cells; *p* = 5 × 10^−8^ by Fisher Exact Test). Notable functions for those genes were related to lipid transport, acyl-CoA metabolism, mitochondrial b-oxidation, and lipases. **d**, **e** qRT-PCR analysis of mRNA expression of CD36 and LPL in HME cell lines; *n* = 3 independent experiments. All values are relative to β-actin and normalised to TERT HME cells. Bars represent mean with upper and lower limits; *p* values were calculated using an unpaired Student’s *t*-test. **f** Measurement of LPL activity in HME cell lysates; *n* = 4 independent experiments. Error bars represents mean ± s.d; *p* values were calculated using an unpaired Student’s *t*-test. **g** Flow cytometric analysis of CD36 surface expression in TERT (grey) and MYC (red) HME cells. Representative histograms from *n* = 2 independent experiments. αGFP (IgG) was used an isotype control (top histogram). **h** Relative mRNA expression of CD36 in MYC HME cells + pTRIPZ, MYC HME cells + shCD36#2, and MYC HME cells + shCD36#3. Cells were treated with 0.5 μg/ml doxycycline for 48 h to induce shRNA expression; *n* = 3 independent experiments. Error bars represent mean with upper and lower limits; *p* values were calculated using an unpaired Student’s *t*-test. **i** Basal OCR of MYC HME cells + pTRIPZ, MYC HME cells + shCD36#2, and MYC HME cells + shCD36#3. Cells were treated with doxycycline to induce shRNA expression prior to measurement of the OCR in the presence of 0.10 mM palmitate; *n* = 5 independent experiments. Error bars represent mean ± standard error of the means (s.e.m.); *p* values were calculated using an ordinary one-way ANOVA with Tukey’s multiple comparisons test; *p < 0.05, **p < 0.01, ***p < 0.001, ****p < 0.0001, N.S. = not significant.

Having established a TNBC model system of isogenic HME cells lines and shown that MYC expression is sufficient to induce FAO in HME cells, we next wanted to identify novel targets that might contribute to the activation of FAO in MYC HME cells. Since MYC is a transcription factor, we performed NextGen RNA sequencing on TERT, MYC, and HER2 HME cells to gain further insight into potential mechanisms of how MYC affects FAO in HME cells (Supplementary Tables S1 and S2). Differentially expressed genes were mapped to networks available in the Ingenuity database. Five networks were identified and ranked by a network score produced by the IPA, that ranged from 30 to 45 (Supplementary Table S3). The score takes into account the number of differentially expressed genes (FDR < 5%) and the size of the network to approximate the relevance of the network to the original list of genes. The only network in MYC HME cells associated with metabolism was “lipid metabolism” (IPA Network 4), whose components are shown in Fig. 2a. The IPA also indicated that some of the most significantly altered canonical signalling pathways (377 genes; FDR < 5%; Min. fold: 6.81) in MYC HME cells were involved in lipid metabolism, including atherosclerosis signalling, LXR/RXR activation, and PPAR signalling (Fig. 2b, black boxes). Gene expression analysis of an unbiased list of 163 different genes (Supplementary Table S4) associated with lipid metabolism across TERT, MYC, and HER2 HME cells revealed a significant enrichment of upregulation in MYC versus TERT (1.4-fold over all upregulated genes, *p* = 5 × 10^−8^ by Fisher Exact Test). Notable functions for those genes were related to lipid transport, acyl-CoA metabolism, mitochondrial β-oxidation, and lipases. Our findings agree with Camarda et al. who also reported a strong representation of fatty acid metabolism genes in TNBC patients.^10^

We next performed a lipidomic analysis that revealed that phosphatidylcholine (PC) and cardiolipin contained longer, more unsaturated fatty acyl chains compared to TERT cells (Supplementary Fig. S2a, b), a pattern commonly observed in cells that are more dependent on fatty acid uptake than de novo fatty acid synthesis.^19,34^ These results also agree with the predicted activation of “atherosclerosis signalling” (Fig. 2b), a process closely associated with enhanced lipid uptake. Furthermore, MYC HME cells contained higher amounts of lipid breakdown products, such as monoacylglycerols (MAGs) and free fatty acids (FFAs), but had 25% less triacylglycerol (TAG), which is the main lipid storage molecule of a cell (Supplementary Fig. S2c). Overall our transcriptomic, bioinformatic, and lipidomic results suggested that MYC HME cells are not simply scavenging fatty acids to store as TAG, but rather actively metabolising them.

### MYC promotes fatty acid uptake and lipase activity in HME cells

We were interested in identifying potential new targets that may be involved in FAO in TNBC. We therefore investigated which specific genes displayed the largest upregulation and were associated with IPA Network 4 and the canonical signalling pathway of atherosclerosis signalling. Fatty acid transporter CD36 (>800-fold upregulation) and LPL (>80-fold upregulation) were two of the most highly upregulated fatty acid metabolism genes in MYC HME cells (Fig. 2a, b and Supplementary Table S1). We confirmed upregulation of CD36 and LPL by qRT-PCR in MYC and T58A HME cell lines (Fig. 2d, e). The physiological function of LPL is to catalyse the release of fatty acids from TAGs that are present in the bloodstream. The liberated fatty acids can then be scavenged by fatty acid transporters, such as CD36. Together, the marked upregulation of LPL and CD36 in MYC HME cells suggested that they may be involved in the mobilisation and consumption of fatty acids in MYC HME cells. MYC HME cells had significantly higher lipase activity compared to TERT or HER2 HME cells (Fig. 2f). We confirmed surface expression of the fatty acid transporter CD36 in MYC HME cells by flow cytometry (Fig. 2g). We then measured fatty acid uptake using time-lapse fluorescent microscopy on TERT and MYC HME cells incubated with BODIPY-labelled dodecanoic acid (BODIPY-C12). MYC HME cells showed higher BODIPY-C12 uptake compared to TERT HME cells (Supplementary Movies S1 and S2).

### Fatty acid uptake through CD36 supports FAO in MYC HME cells

We predicted that CD36-mediated fatty acid uptake may be involved in the ability of MYC HME cells to oxidise fatty acids. We generated stable pools of MYC HME cells that express two different TetON-inducible shRNAs against CD36 (Fig. 2h). shRNA-mediated silencing of CD36 expression inhibits FAO in MYC HME cells, suggesting that CD36 is a major contributor to the import of fatty acids that are destined for oxidation in the mitochondria (Fig. 2i).

Multiple studies have suggested that mitochondria-localised carnitine acyltransferases, such as CPT1A and CPT2, are important regulators of FAO in breast cancer and critical for breast cancer growth.^10,35,36^ MYC and T58A HME cells had ~2-fold increases in CPT mRNA expression compared to TERT and HER2 (Supplementary Fig. S2d, e). LC-MS-based metabolomics analysis showed that MYC and T58A HME cells also have significantly higher uptake of carnitine, which is a substrate of CPT1A and a necessary amino acid for long-chain fatty acid import into mitochondria (Supplementary Fig. S2f). We next measured the levels of and labelling of acetyl-carnitine, a metabolite closely linked to FAO and whose increased presence suggests active FAO.^37,38^ Acetyl-carnitine is synthesised from acetyl-CoA in the mitochondria by the enzyme carnitine acetyltransferase. Since acetyl-carnitine is an “end product” that cannot be further metabolised, it can only be converted back into acetyl-CoA. It is therefore important to take into account the total level of acetyl-carnitine in the cell when measuring acetyl-carnitine synthesis. Although there were differences between TERT and MYC HME cells with respect to which fuel was used for acetyl-carnitine synthesis, when we added together the percent labelling of acetyl-carnitine by ^13^C-glucose, ^13^C-palmitate, and ^13^C-gutamine, there were no differences between MYC HME cells (16.46%) and TERT HME cells (16.74%) (Supplementary Fig. S2g–i). However, we did find that the total mitochondrial acetyl-carnitine level was >4 times higher in MYC HME cells compared to TERT HME cells (Supplementary Fig. S2j–l), suggesting a much higher rate of acetyl-carnitine production in MYC HME cells. In addition, we detected efflux of acetyl-carnitine into the medium from MYC and T58A HME cells, but not TERT and HER2 HME cells, further evidence of highly active FAO in MYC HME cells (Supplementary Fig. S2m). Overall, these results suggest that MYC causes increased fatty acid uptake, increased carnitine uptake, and increased FAO in HME cells and identified CD36 as a potential mediator of fatty acid uptake and oxidation in MYC HME cells.

### CAMKK2 phosphorylates AMPK in MYC HME cells

**Fig. 3:**
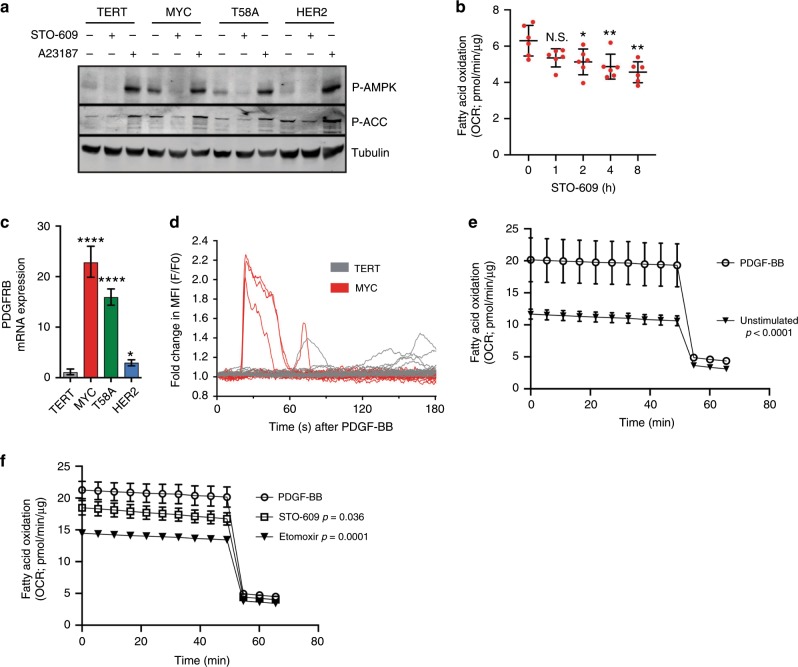
Ca^2+^ dependent activation of CAMKK2 promotes fatty acid oxidation. **a** P-AMPK and P-ACC expression after inhibition of CAMKK2 kinase activity by STO-609 (10 μM) or stimulation of CAMKK2 by the Ca^2+^ ionophore A23187 (1 μM). **b** Basal OCR of MYC HME cells after pre-incubation in STO-609 (10 μM). The OCR was measured in the presence of 0.10 mM palmitate; *n* = 5 replicates from one independent experiment. Error bars represent mean ± s.e.m.; *p* values were calculated using an ordinary one-way ANOVA and Tukey’s multiple comparison test. **c** Relative mRNA expression of PDGFRB in HME cells normalised TERT and relative to β-actin; *n* = 3 independent experiments. Error bars represent mean with upper and lower limits; *p* values were calculated using an unpaired Student’s *t*-test. **d** Fold change in intracellular [Ca^2+^] in TERT and MYC HME cells upon PDGF-BB treatment. Traces represent individual cells for each cell line. **e** Basal OCR of MYC HME cells in the absence (unstimulated) and presence of 25 ng/ml PDGF-BB. All OCR measurements were done in the presence of 0.10 mM palmitate; *n* = 5 replicates. Error bars represent mean ± s.e.m. Total oxygen consumption was calculated based on the area under the curve for each sample. The average of total oxygen consumption was compared between groups using ANOVA with post-hoc multiple comparisons. Unadjusted *p* values are reported. **f** Basal OCR of MYC HME cells after treatment with STO-609 (10 μM) or etomoxir (40 μM). All OCR measurements were done in the presence of 0.10 mM palmitate; *n* = 5 replicates. Error bars represent mean ± s.e.m. Total oxygen consumption was calculated based on the area under the curve for each sample. The average of total oxygen consumption was compared between groups using ANOVA with post-hoc multiple comparisons. Unadjusted *p* values are reported; *p < 0.05, **p < 0.01, ***p < 0.001, ****p < 0.0001, N.S. = not significant.

The increase in the expression of fatty acid import machinery at both the plasma membrane by CD36 and inner mitochondrial membrane by CPT1A and CPT2 facilitates increased uptake and FAO in MYC HME cells. However, one of the main mechanisms by which FAO is regulated is through 5′-AMP-activated protein kinase (AMPK)-dependent phosphorylation of acetyl-CoA carboxylase (ACC). ACC produces malonyl-CoA, a potent inhibitor of CPT1A. Inhibition of ACC by AMPK phosphorylation therefore alleviates CTP1A inhibition to stimulate FAO. Camarda et al. showed that TNBC, compared to receptor-positive breast cancer, has lower expression of fatty acid synthase (FASN) and ACC and that this lower expression was associated with worse prognosis.^10^ Although we did not observe downregulation of FASN or ACC in MYC HME cells, we did find that MYC expression caused increased AMPK phosphorylation in HME cells (Fig. 3a and Supplementary Fig. S3a). AMPK is typically phosphorylated in response to nutrient stress, such as an increase in the AMP:ATP ratio. However, metabolomic analysis indicated that the AMP, ADP, and ATP concentrations are not different between TERT and MYC HME cells, ruling out a role for ATP stress in AMPK phosphorylation (Supplementary Fig. S3b).

There is a second known AMPK kinase called calcium (Ca^2+^)/calmodulin activated kinase kinase 2 (CAMKK2) that can phosphorylate AMPK in a Ca^2+^/calmodulin (CaM)-dependent manner.^39^ We observed a ~2-fold increase in CAMKK2 mRNA and protein expression in MYC and T58A HME cells (Supplementary Fig. S3c, d). Inhibition of CAMKK2 by a pharmacological inhibitor (STO-609) or siRNA-mediated silencing of CAMKK2 strongly inhibited AMPK phosphorylation in MYC and T58A HME cells (Fig. 3a and Supplementary Fig. S3e). Conversely, activation of CAMKK2 using a Ca^2+^ ionophore (A23187) causes a robust increase in the phosphorylation of AMPK (Fig. 3a). Altogether these findings suggest that activation of AMPK occurs independently of energy stress and is regulated by CAMKK2 in MYC HME cells. Importantly, STO-609 caused a time-dependent decrease in FAO in MYC HME cells, thereby suggesting that CAMKK2 activity helps to promote FAO (Fig. 3b).

### Ca^2+^ signalling via CAMKK2 helps to promote FAO

When Ca^2+^ binds to CaM, it increases the affinity of CaM for CAMKK2. Binding of the Ca^2+^ bound CaM to CAMKK2 induces autophosphorylation and full activation of CAMKK2. We therefore explored the importance of Ca^2+^ in CAMKK2 activation and found that chelation of intracellular Ca^2+^ by BAPTA-AM causes a decrease in the phosphorylation of AMPK, suggesting that Ca^2+^ signalling is involved in the CAMKK2-dependent phosphorylation of AMPK (Supplementary Fig. S3f). This result also suggested that MYC HME cells may have altered Ca^2+^ signalling. To further investigate this, we compared Ca^2+^ signalling in TERT and MYC HME cells by pre-loading them with the intracellular Ca^2+^ indicator Fluo-4 and caged IP_3_. Caged IP_3_ is inert until a pulse of ultraviolet (UV) light is given to release the IP_3_, which then allows comparison of IP_3_-induced Ca^2+^ release in TERT and MYC HME cells. The Ca^2+^ release in response to IP_3_ uncaging in MYC HME cells was extremely fast and often occurred so quickly that it was buried within the flash artefact of the UV pulse (Supplementary Fig. S3g). We then quantified the time from uncaging to the half maximal peak of Ca^2+^ release and found that MYC HME cells rapidly respond to IP_3_ as opposed to TERT cells, which exhibited a significant delay (Supplementary Fig. S3g, h).

The caged-IP_3_ experiments suggested changes to intracellular Ca^2+^ handling in MYC HME cells, we therefore mined the RNA sequencing data to identify potential modulators of Ca^2+^ signalling. We found that MYC and T58A HME cells have much lower expression of three major Ca^2+^ transporters: the Na^+^/Ca^2+^ exchanger SLC8A1, a golgi-resident Ca^2+^ pump ATP2C2, and a plasma membrane Ca^2+^ ATPase ATP2B4 (Supplementary Fig. S3i–k, Supplementary Table S1). Interestingly, depletion of MYC by siRNA resulted in increased expression of Ca^2+^ transporters suggesting that MYC may modulate Ca^2+^ handling as part of a mechanism by which it regulates cell metabolism (Supplementary Fig. S3l, m).

We also identified significant upregulation of PDGFRB in MYC HME cells (Fig. 3c and Supplementary Fig. S3n). PDGFRB is a receptor tyrosine kinase (RTK) that induces intracellular Ca^2+^ release and has strong links to epithelial-to-mesenchymal transition (EMT), cell migration, and cancer progression.^40,41^ To assess the role of PDGFRB in the activation of FAO, we loaded MYC and TERT HME cells with the Ca^2+^-binding dye Fluo-3 and measured intracellular Ca^2+^ release in response to “puffing” its ligand, PDGF-BB, onto the cells (as previously described in ref. ^42^). In the MYC HME cells that responded (3/18), there is a strong rise in cytosolic Ca^2+^ concentration ([Ca^2+^]_c_) with a mean fold change of 2.16 ± 0.12 (Fig. 3d). We also noted that one of the initial responders displayed a second [Ca^2+^]_c_ peak, suggesting an oscillation in Ca^2+^ release (Fig. 3d). TERT HME cells that responded (5/29) showed a later (from 60 s onwards), modest rise in [Ca^2+^]_c_ (1.32 ± 0.11) (Fig. 3d). Overall, TERT HME cells have a delayed, weak response to PDGF-BB while MYC HME cells had Ca^2+^ rises that were faster, higher, and sustained for longer in response to PDGF-BB stimulation.

We next tested if PDGFRB stimulation was able to promote FAO in MYC HME cells. A 1-h preincubation of MYC HME cells in the presence or absence of PDGF-BB demonstrated that PDGFRB signalling causes a large increase in FAO (Fig. 3e). PDGF-BB-induced FAO could be inhibited by STO-609 or etomoxir (Fig. 3f). STO-609 treatment in the absence of growth factor did not have a substantial effect on FAO after 1-h preincubation (Fig. 3b), suggesting the effects were specific to the PDGFRB response. Etomoxir treatment in the absence of growth factors suppressed FAO (Supplementary Fig. S3o); however, this was expected since the cells likely engage in some degree of FAO in the absence of stimulation. Interestingly, these results were also seen when we stimulated a different RTK, EGFR, with EGF (Supplementary Fig. S3p, q). Altogether, these results suggest that RTK-mediated Ca^2+^ release and subsequent stimulation of CAMKK2 is one way by which MYC expressing HME cells may promote FAO.

### CAMKK2 inhibition has cytostatic and cytotoxic effects on MYC HME cells

**Fig. 4:**
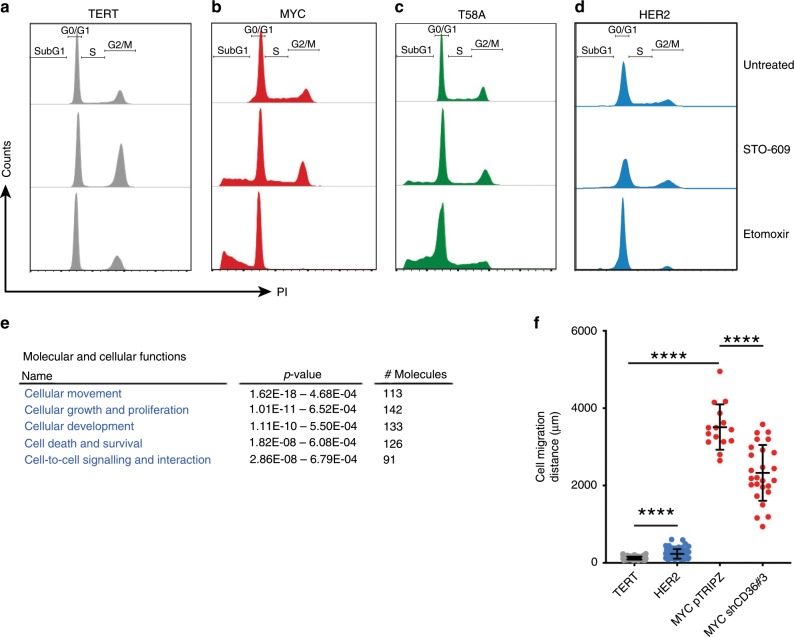
Fatty acid oxidation promotes cell survival and migration. **a–d** Cell cycle analysis of HME cell lines treated with STO-609 (10 μM) or etomoxir (150 μM) for 48 h. Data are representative of one of *n* = 2 independent experiments. **e** Table highlighting the most significantly altered molecular and cellular functions in MYC HME cells compared to TERT HME cells based on RNA sequencing data in Supplementary Table S1 and Ingenuity Pathway Analysis. **f** Total distance moved by TERT, HER2, MYC+pTRIPZ and MYC+shCD36#3 HME cells over an 8-h period; *n* = 3 independent experiments. Each dot represents an individually tracked cell. Error bars represent mean ± s.e.m; *p* values were calculated using an ordinary one-way ANOVA Tukey’s multiple comparison test; *p < 0.05, **p < 0.01, ***p < 0.001, ****p < 0.0001, N.S. = not significant.

We next wanted to determine the long-term effects of inhibiting CAMKK2 signalling on proliferation and survival of MYC HME cells. STO-609 caused cytostatic effects in MYC and T58A HME cells as evidenced by decreases in the percentage of cells in S and G2/M phases of the cell cycle (Fig. 4a–d and Supplementary Fig. S4a). STO-609 also caused cell death in 15–20% of MYC HME cells (Fig. 4a–d and Supplementary Fig. S4a). Etomoxir, a potent FAO inhibitor, caused substantial cell death in MYC and T58A HME cells. In contrast, TERT and HER2 HME cells had little to no cell death in response to STO-609; however, there were decreases in S phase and large increases in G2/M phase of the cell cycle. Other than a decrease in the percentage of S phase cells, TERT and HER2 HME cells were largely unaffected by treatment with etomoxir.

### Inhibition of CAMKK2 or CD36 strongly reduces MYC HME cell migration

From the previous time-lapse movies of BODIPY-C12 uptake, we noticed that MYC HME cells were highly migratory compared to TERT HME cells (Supplementary Movies 1 and 2). In addition, IPA predicted “cellular movement” as the top hit for molecular and cellular functions (*p* = 1.62e−18; Fig. 4e). When we tracked the migration of individual cells using time-lapse microscopy, we measured a striking 10-fold increase in MYC HME cell migration speeds compared to TERT or HER2 cells (Fig. 4f). Depletion of CD36 or inhibition of CAMKK2 significantly impaired MYC HME cell migration (Fig. 4f and Supplementary Fig. S4b). These results suggest that CAMKK2 and CD36 are important contributors to cell migration.

### The fatty acid metabolism gene signature of MYC HME cells is highly representative of claudin-low TNBC

**Fig. 5:**
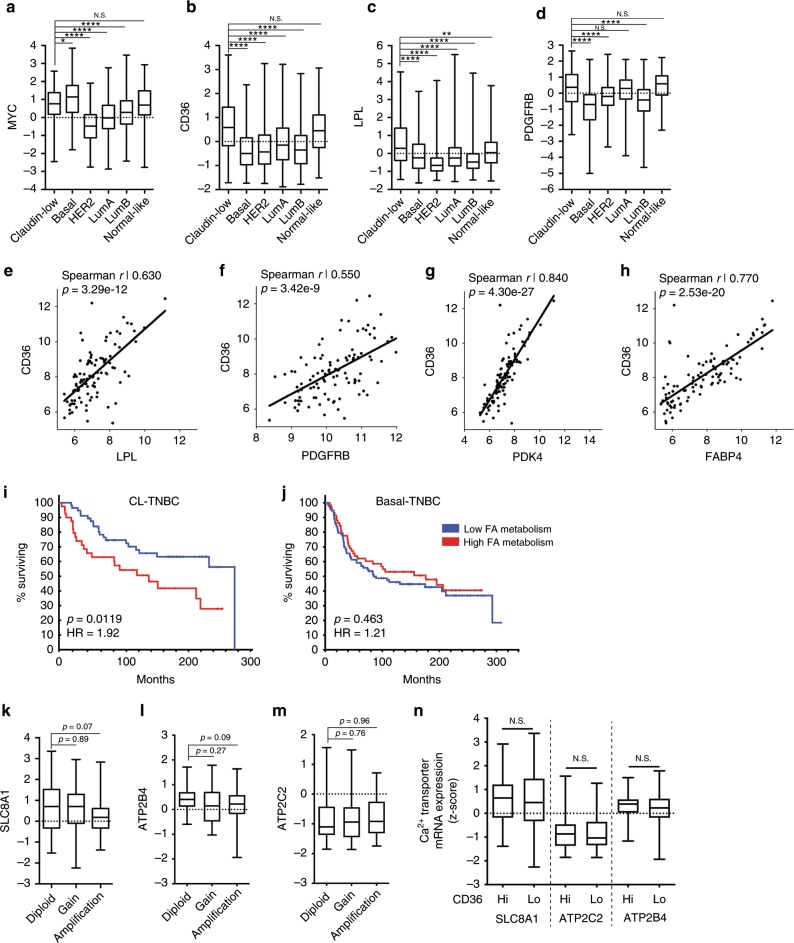
The fatty acid metabolism gene expression signature of MYC HME cells is highly representative of human claudin-low breast tumours. **a–d** Box and whisker plots of fatty acid metabolism gene mRNA expression levels (*z*-scores) in human breast cancer patient tumours from the METBRIC database. Tumours were classified based on the PAM50 + claudin-low molecular subtyping of breast cancer as called by the METABRIC database. Claudin-low, *n* = 182; Basal, *n* = 198; HER2, *n* = 218; Luminal A, *n* = 673; Luminal B, *n* = 454; Normal-like, *n* = 135. Boxes represent 25th to 75th percentile, whiskers represent min to max, and lines represent medians; *p* values were determined using an ordinary one-way ANOVA and Dunnett’s multiple comparison test. **e–h** Scatter plots of mRNA expression of CD36 against LPL, PDGFRB, PDK4, and FABP4 in CL-TNBC. Black lines represent linear regression of scatter plots. Spearman correlation *r* values and *p* values are reported on graphs. **i**, **j** Kaplan–Meier survival plots of patients with basal-TNBC (**j**) and CL-TNBC (**i**). Patients were stratified based on the expression of CD36, LPL, PDK4, and FABP4 as described in the results; *p* values were calculated using log-rank test. Hazard ratios (HR) are shown on the plots. **k–m** Box and whisker plots of Ca^2+^ transporter mRNA expression levels (*z*-scores) in CL-TNBC patient tumours. Tumours were divided in those with diploid, copy number gains, and amplification of *MYC* as called by the METBRIC database. Boxes represent 25th to 75th percentile, whiskers represent min to max, and lines represent medians; *p* values are reported on the graphs and were determined using an ordinary one-way ANOVA and Dunnett’s multiple comparison test. **n** Box and whisker plots of Ca^2+^ transporter mRNA expression levels (*z*-scores) in CD36^Hi^ versus CD36^Lo^ CL-TNBC patient tumours. Boxes represent 25th to 75th percentile, whiskers represent min to max, and lines represent medians; *p* values were determined using an unpaired Student’s *t*-test; *p < 0.05, **p < 0.01, ***p < 0.001, ****p < 0.0001, N.S. = not significant.

We had thus far found that expression of oncogenic levels of MYC in human breast cells induces a multigenic program characterised by changes in RTK signalling, intracellular Ca^2+^ handling, CAMKK2 signalling, fatty acid uptake, and FAO (Supplementary Fig. S4c). We next sought to determine if the fatty acid metabolism gene signature of the MYC HME cells is representative of specific molecular subtype of breast cancer. Since MYC and T58A HME cells lack ER, PR, and HER2 expression (Fig. 1a, b), we predicted that their gene expression patterns would most closely reflect TNBC. Bioinformatic analysis of the METABRIC breast cancer database of ~2000 patient tumour samples showed that MYC is indeed most highly expressed in claudin-low (CL) and basal breast cancers, which are also the two subtypes most highly enriched for TNBCs (Fig. 5a).^1,2,23,24^ However, CL breast cancers specifically had the highest expression of CD36, LPL, and PDGFRB (Fig. 5b–d). We next compared the expression of MYC, CD36, LPL, and PDGFRB in basal-TNBC versus CL-TNBC. Although we did not observe a significant difference in MYC (*p* = 0.08) or LPL (*p* = 0.11) expression between basal-TNBC and CL-TNBC, we did find that CL-TNBC tumours had significantly higher expression of CD36 (*p* < 0.0001) and PDGFRB (*p* < 0.0001) (Supplementary Fig. S5a–d).

There was significant correlation in expression between CD36 and LPL (Spearman 0.63; *p* = 3.29e−12) and CD36 and PDGFRB (Spearman 0.55; *p* = 3.42e−9), suggesting that high expression of these genes often co-occurs in CL-TNBC (Fig. 5e, f). According to the METABRIC database, the two genes with the highest degree of co-expression with CD36 were pyruvate dehydrogenase kinase 4 (PDK4; Spearman 0.84; *p* = 1.73e−17) and fatty acid binding protein 4 (FABP4; Spearman 0.77; *p* = 2.27e−21) (Fig. 5g, h). Similar to CD36, PDK4 and FABP4 are also highly expressed in CL breast cancers compared to all other molecular subtypes with the exception of normal-like breast tumours. Furthermore, when we compared the expression of PDK4 an FABP4 in basal-TNBC versus CL-TNBC, we again found significantly higher expression of PDK4 (*p* < 0.0001) and FABP4 (*p* < 0.0001) in CL-TNBC (Supplementary Fig. S5e–h).

PDK4 is a negative regulator of glucose oxidation that phosphorylates and thereby inhibits pyruvate dehydrogenase activity. There is extensive literature on the role of PDK4 in stimulating mitochondrial FAO.^43,44^ PDK4 is often thought of as a molecular switch between glucose oxidation and FAO, such that high PDK4 activity promotes FAO.^43^ Interestingly, it has been shown that fatty acid influx and enforced expression of CD36 are able to induce the expression of PDK4 in muscle suggesting a strong metabolic link between the two.^45^ Knockdown of PDK4 impacts breast cancer metabolism and high PDK4 expression has been shown to predict poorer survival in breast cancer patients.^46^ FABP4 has been shown to promote the direct transfer of lipids from adipocytes to ovarian cancer cells that then promotes in vitro and in vivo tumour growth.^47^ A more recent study showed that exogenous expression of FABP4 in CL-TNBC cells (MDA-MB-231) increased proliferation.^48^ Interestingly, *FABP4* is on the same arm of chromosome 8 as *MYC* and is often co-amplified with *MYC* in breast cancer (Supplementary Fig. S5i, j). Since we used exogenous expression of MYC to increase MYC expression in HME cells, this may explain why FABP4 was not upregulated in MYC HME cells (Supplementary Table S1).

We created a “fatty acid metabolism gene signature” consisting of CD36, LPL, PDK4, and FABP4 and checked each molecular subtype of breast cancer for upregulation or amplification of these genes. If any one or combination of these genes was upregulated or amplified in the tumour, the patient was classified as “high fatty acid metabolism”. All patient tumours that lacked upregulation or amplification of all four of these genes were classified as “low fatty acid metabolism”. We next tested if this four gene fatty acid metabolism gene signature predicts poorer survival only in TNBC or in all breast cancer subtypes. Despite consistent correlation in expression between CD36, LPL, PDK4, and FABP4 in all breast cancer subtypes (data not shown), the only subtype that displayed poorer survival was CL-TNBC (*p* = 0.0119; HR = 1.92; Fig. 5i, j and Supplementary Fig. S5k–p). We performed a Cox regression analysis using the fatty acid metabolism gene signature and age and found that both are independent predictors of survival (data not shown). Our bioinformatic analyses suggested that this set of fatty acid metabolism genes and, by extension, FAO may be associated with more aggressive disease in CL-TNBC, but not necessarily other subtypes of TNBC (Fig. 5g, h and Supplementary Fig. S6h–m).

We next compared the expression of SLC8A1, ATP2C2, and ATP2B4 with respect to *MYC* gene amplification in CL-TNBC. Although it did not reach significance, SLC8A1 and ATP2B4 did have lower expression levels in CL-TNBC tumours that have *MYC* gene amplification (*p* = 0.07 and *p* = 0.09, respectively) (Fig. 5k, l). No differences in expression were noted for ATP2C2 with regard to *MYC* gene amplification in CL-TNBC (Fig. 5m). We then tested if CD36^Hi^ tumours had lower expression of Ca^2+^ transporters. We did not detect any differences in Ca^2+^ transporter expression in CD36^Hi^ versus CD36^Lo^ CL-TNBC tumours (Fig. 5n).

### MYC expression in HME cells induces EMT

The fact that basal-TNBC and CL-TNBC tumours had similar expression levels of MYC yet very different expression levels of CD36, LPL, PDK4 and FABP4 suggested that there are likely other factors, besides MYC, that direct activation of FAO and lipid metabolism in CL-TNBC. Furthermore, the lack of correlation between CD36 expression and Ca^2+^ transporter expression also suggested that MYC may not directly modulate lipid metabolism gene expression. We therefore tested the impact of siRNA-mediated silencing of MYC on expression of CD36, LPL and PDK4. Knockdown of MYC repressed expression of LPL and PDK4, but not CD36, suggesting an alternative regulator of CD36 expression in MYC HME cells (Supplementary Fig. S6a–d).

We re-examined the RNA sequencing data for potential modulators of CD36. Elevated fatty acid uptake via CD36 has been shown to promote EMT in hepatocellular caricinoma^49^ and EMT is a defining characteristic of CL-TNBC.^29^ Interestingly, one of the top canonical signalling pathways in MYC HME cells was “Regulation of the EMT pathway” (Fig. 2b; red box). In addition, the top scoring network from the IPA was “embryonic and organismal development” (IPA network 1) (Supplementary Fig. S7a). IPA Network 1 suggested downregulation of E-cadherin (CDH1), Wnt signalling, and FOXF2 (Supplementary Fig. S7a, black circles). FOXF2 suppresses EMT in breast cancer and is inversely correlated with the EMT activator FOXC2.^50,51^ We used qRT-PCR to confirm downregulation of FOXF2 and upregulation of FOXC2 (Supplementary Fig. S7b, c). MYC HME cells additionally have high expression of other EMT inducers, such as ZEB1, ZEB2, and TWIST1 (Supplementary Figs. S6e,  S7d, e, and Supplementary Table S1). We also confirmed downregulation of cell adhesion molecules, including E-cadherin (CDH1) and claudin 4 (CLDN4), another defining characteristic of CL-TNBC (Supplementary Fig. S7f, g and Supplementary Table S1).^29^ Given the presence of an EMT gene signature in the MYC HME cells, we hypothesised that EMT inducers might promote CD36 expression. siRNA-mediated knockdown of TWIST1, a transcription factor that promotes EMT, decreased CD36 expression by 50% suggesting that expression of CD36 in MYC HME cells may be linked to the process of EMT (Supplementary Fig. S6f, g). The expression of TWIST1 and CD36 highly correlates in CL-TNBC further suggesting these two molecules may be functionally linked (Supplementary Fig. S6h).

### MYC HME cells possess many of the characteristics of mesenchymal stem-like TNBC

IPA Network 1 also suggested activation of hedgehog (Hh) signalling (Supplementary Fig. S7a). Hh signalling plays an important role in embryogenesis, stem-cell renewal, and EMT.^52^ The glioma-associated oncogene family, which includes GLI2, are the master transcriptional regulators of Hh signaling.^53,54^ We therefore revisited the RNA sequencing data to identify other genes that contribute to the stem-like phenotype associated with IPA Network 1. Besides MYC itself, we find upregulation of other breast cancer stem cell factors (SOX2, SOX18, THY1, EYA1, GLI2, SALL1, GLIS1, CXCR4), suggesting that MYC HME cells adopt a stem-like state (Supplementary Fig. S6i–l and Supplementary Table S1). Consistent with this result, MYC HME cells had little to no surface expression of CD24, a surface marker commonly lost on cancer stem cells (also called tumour initiating cells) (Supplementary Fig. S6m).^55,56^ It was reported that the gene expression profile of CL-TNBC tumours closely clusters with that of so-called “mesenchymal stem-like” tumours. Mesenchymal stem-like tumours have a high degree of dedifferentiation, low expression of claudins, loss of E-cadherin and CD24, and high expression of genes associated with EMT, including PDGFRB and TWIST.^57^ Furthermore, PDGFR signalling is known to induce EMT in breast cancer.^58^ Altogether, the major transcriptional changes that occurred in MYC HME cells are also the same changes that occur in CL-TNBC. The data also suggest that acquisition of an EMT phenotype in the presence of oncogenic levels of MYC may be necessary for robust activation of FAO and serve as a distinguishing feature for differentiating CL-TNBC from basal-TNBC.

### CL-TNBC cell lines are sensitive to STO-609 and etomoxir

**Fig. 6:**
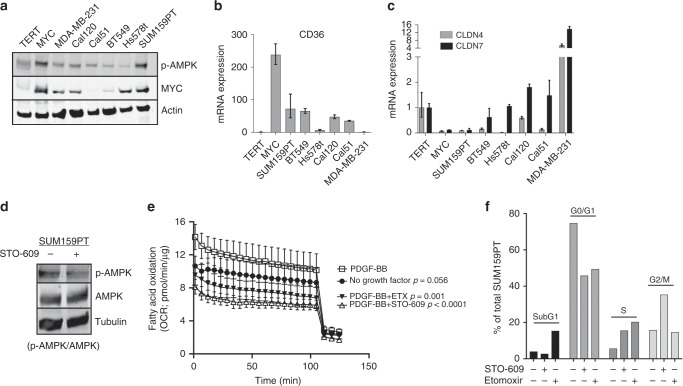
Claudin-low triple negative breast cancer cell lines possess similar characteristics to MYC HME cells and are sensitive to STO-609 and etomoxir. **a** Immunoblot analysis for protein expression of MYC and p-AMPK in a panel of TNBC cell lines, including TERT and MYC HME cells. **b**, **c** Relative mRNA expression of CD36 (**b**), CLDN4 (**c**, grey bars), and CLDN7 (**c**, black bars) in a panel of TNBC cell lines, including TERT and MYC HME cells. Data are normalised TERT and relative to β-actin; *n* = 3 independent experiments. Error bars represent mean with upper and lower limits. **d** Inhibition of CAMKK2 by STO-609 inhibits phosphorylation of AMPK in SUM159PT, a CL-TNBC cell line. **e** Basal OCR of SUM159PT cells ± PDGF-BB (25 ng/ml) stimulation and treatment with STO-609 (10 μM) or etomoxir (40 μM). All OCR measurements were done in the presence of 0.10 mM palmitate; *n* = 5 replicates. Error bars represent mean ± s.d. Total oxygen consumption was calculated based on the area under the curve for each sample. The average of total oxygen consumption was compared between groups using ANOVA with post-hoc multiple comparisons. **f** Flow cytometry analysis of cell cycle using propidium iodide. Both cell cycle and cell survival (subG1) of SUM159PT cells are markedly affected by inhibition of CAMKK2 by STO-609 (10 μM) and blockade of FAO by etomoxir (150 μM). Unadjusted *p* values are reported; *n* = 1 independent experiment.

Given the similarities between CL-TNBC and MYC HME cells, we analysed a panel of TNBC cell lines for expression of p-AMPK, MYC, CD36, CLDN4, and CLDN7. SUM159PT cells expressed MYC to a similar level as MYC HME cells, had the highest expression of CD36 and p-AMPK among the TNBC cell lines, and had complete loss of both claudin 4 and claudin 7 (Fig. 6a–c). Treatment of SUM159PT cells for two hours with STO-609 led to a decrease in p-AMPK levels, suggesting that activation of AMPK occurs in a CAMKK2-dependent manner in SUM159PT cells (Fig. 6d). We also saw decreased phosphorylation of AMPK in response to STO-609 in two other CL-TNBC cell lines (Supplementary Fig. S8a). We next tested the ability of PDGF-BB to stimulate FAO in SUM159PT cells and indeed we observed increased FAO upon treating SUM159PT cells with PDGF-BB (Fig. 6e). This increase in FAO could be blocked by the addition of STO-609 or etomoxir. STO-609 also causes an increase in the percentage of SUM159PT cells in S and G2/M phases of the cell cycle, while etomoxir induced cell death and an accumulation in S phase (Fig. 6f). In sum, these results suggest that it may be possible to target FAO in CL-TNBC.

## Discussion

Recent studies have highlighted a role for FAO in the growth of TNBC and suggested that targeting FAO may be beneficial to TNBC patients.^10,13,14,59^ The activation of FAO in TNBC has been linked to overexpression of the oncogene *MYC*, and previous studies have shown that inhibition of MYC increases lipid droplet formation in tumours by causing mitochondrial dysfunction.^10,60,61^ However, the mechanistic underpinnings by which MYC stimulates FAO in TNBC have remained elusive. We therefore set out to obtain a deeper understanding of how MYC regulates the import and oxidation of fatty acids in TNBC.

We found that the activation of FAO is facilitated by upregulation of LPL and CD36 which mobilise and scavenge fatty acids. MYC HME cells consumed substantially more carnitine and harboured large stores of acetyl-carnitine, a high energy by-product of FAO. We also found that the overall metabolic flux of two major mitochondrial fuel sources (i.e. glutamine and fatty acids) was higher in MYC HME cells compared to TERT HME cells. CD36 facilitates increased fatty acid uptake and in combination with PDK4-mediated inhibition of pyruvate dehydrogenase would promote FAO and inhibit glucose oxidation. Inhibition of glucose oxidation by PDK4 thus allows CL-TNBC cells to use fatty acids to fuel mitochondrial acetyl-CoA production which, in turn, might spare pyruvate and upstream glycolytic intermediates for other biosynthetic pathways. Such a metabolic transformation would be highly advantageous to highly proliferative and highly migratory cancer cells.

Based on our findings, we also propose that Ca^2+^ release from internal stores stimulates the activation of CAMKK2 and AMPK to further promote FAO. We suggest that RTKs such as PDGFRB and EGFR, both of which are highly expressed in TNBC, activate intracellular Ca^2+^ release. However, it is possible that other RTKs or G-protein coupled receptors may also be involved. MYC may also further potentiate Ca^2+^ signalling by repressing expression of Ca^2+^ transporters, thereby compromising the ability of the cell to efficiently export Ca^2+^ from the cytosol. MYC-induced downregulation of the plasma membrane Ca^2+^ pump ATP2B4 did impact Ca^2+^ efflux in B cells and has been linked to proliferation and differentiation of B cells.^62^ Our study is the first to show that Na^+^/Ca^2+^ exchanger SLC8A1 is repressed by MYC. Downregulation of the Na^+^/Ca^2+^ exchanger SLC8A1 has been associated with apoptosis evasion in penile carcinomas.^63^ Our results highlight that further studies on the impact of ion channels and Ca^2+^ signalling on cancer metabolism are needed.

Increases in AMPK phosphorylation occur upon induction of MYC in osteosarcoma cells^64^ and a recent report suggested that Ca^2+^ signalling is involved in MYC-associated activation of NUAK1, a member of the AMPK-related family of kinases.^65^ Furthermore, CAMKK2 is upregulated and phosphorylates AMPK in prostate cancer, a cancer type that has high MYC expression.^66–69^ ChIPseq analysis has shown that MYC binds to the CAMKK2 promoter region.^70^ We found that inhibition of CAMKK2 blocked AMPK phosphorylation, inhibited PDGF-induced FAO, and caused cytostatic and cytotoxic effects on MYC HME cells and SUM159PT CL-TNBC cells. The alterations to the cell cycle distribution caused by STO-609 suggested that there may be other CAMKK2 functions, outside of stimulating FAO, that control cell cycle. In support of this concept, siRNA-mediated silencing of CAMKK2 expression in prostate cancer cells has been shown to increase the percentage of cells in G1 phase.^71^

MYC and TERT HME cells have similar proliferation rates (data not shown), but the cell migration velocity of MYC HME cells was >10-fold higher than TERT HME cells. We propose that the heightened metabolic state induced by MYC expression is able to satisfy the high energy demands of cells that are both highly migratory and highly proliferative. It is tempting to speculate that MYC accomplishes this task by “hijacking” Ca^2+^ signalling as a means to activate AMPK during otherwise nutrient replete conditions. By using Ca^2+^ to activate AMPK, MYC HME cells create a pseudo starvation state of metabolic stress. AMPK would then further stimulate multiple metabolic pathways, including FAO. This boost in energy production would provide an advantage to TNBC by supporting energy demanding process of cell migration while also allowing cancer cells to satisfy the biomass and energy demands of proliferation. Indeed, we found that inhibition of CD36 or CAMKK2 decreased cell migration.

We envision that migrating cells require a high level of energy production that CD36 mediated fatty acid uptake and FAO can provide. Our lipidomic analysis showed that the fatty acids being taken up by MYC HME cells were also being used for lipid biosynthesis suggesting that CD36 may also contribute to membrane biogenesis. The intricacies of membrane remodelling associated with pseudopod extension and cell surface projections remains controversial and further studies are needed to elucidate the exact changes in the types and composition of phospholipids that reside at leading edges of migrating and invading cells.^72^ Interestingly, CD36 is an emerging metabolic target and has been proposed to be crucial for cell migration, invasion, and metastasis of oral squamous cell carcinoma,^73^ gastric cancer,^74^ and ovarian cancer.^75^ Our results also agree with a study that suggested that mobilisation of fatty acids from adipocytes promotes breast cancer invasion.^76^ FABP4, which is often co-amplified with MYC and almost always highly co-expressed with CD36 in breast cancer, was shown to be critical for ovarian cancer metastasis to the omental fat pad where it supported fatty acid transport and FAO.^47^

Perhaps the most intriguing finding from our study was that CL-TNBC, specifically, had much higher expression of CD36, LPL, PDGFRB, FABP4, and PDK4 compared to all other breast cancers, including basal-TNBC. Given the associations between these fatty acid metabolism genes and FAO, we suggest that CL-TNBC patients are likely to experience the greatest benefit from targeting FAO. There are currently no routine diagnostic tools for identifying CL breast cancer in the clinic and gene expression profiling remains the gold standard for molecular subtyping of TNBC.^77^ As such, CL breast cancer is often more broadly categorised as TNBC in the clinic and targeted therapies are non-existent for patients with CL breast cancer.^78^ Efforts are already underway to better diagnose CL breast cancer in the clinic and it will be interesting to see the potential of fatty acid metabolism genes to serve as markers of CL breast cancer.

Our research also supports a rationale for inhibiting one or more of the alternative FAO targets identified in our study, such CAMKK2 and CD36. Targeting CAMKK2 in combination with CD36 could impact FAO at two different points, and the secondary functions of these targets in cell cycle regulation and membrane biogenesis, respectively, may offer further benefit as well. Overall, our study provides a deeper understanding of the link between TNBC and FAO and shows that MYC promotes a multigenic program that drastically changes the behaviour and metabolism of breast cells.

Our findings have the potential to impact breast cancer treatment beyond CL-TNBC. For instance, it has been suggested that regardless of the initial breast cancer subtype, the cancer cells remaining after chemotherapy or endocrine therapy (i.e. relapsed tumours) largely adopt CL breast cancer characteristics.^79^ Furthermore, it is interesting to note that many mouse models of breast cancer eventually adopt CL breast cancer characteristics.^80,81^ An example is that inactivation of MYC expression in breast tumour bearing mice leads to tumour regression, but many of these tumours eventually recur.^81^ Unsupervised gene clustering analysis of these recurrent breast tumours indicated enrichment for a CL gene signature, which was completely absent in the primary tumours. Likewise, ~20% of the tumours that emerge in MMTV-MYC model adopt an EMT/squamous tumour phenotype that is highly similar to claudin-low breast cancer.^80^ This phenomenon, however, is not exclusive to MYC-driven models. Recurrent Neu-induced (i.e. HER2+) mammary tumours also display an EMT phenotype.^82^ There is, therefore, a crucial need to identify novel therapeutic opportunities in CL breast cancer, not only because it is a poorly understood molecular subtype, but also because its gene expression patterns are highly representative of a large proportion of resistant and recurrent tumours in human breast cancer patients.

Our bioinformatic analyses revealed striking similarities between these aforementioned mouse studies and our MYC HME cells. MYC HME cells adopted a CL-TNBC gene signature, but interestingly many of the EMT associated genes were not directly regulated by MYC (i.e. CD36, ZEB1, ZEB2, PDGFRB, etc). It is becoming clearly evident that cancer cells can evolve and thrive independent of the oncogene-initiating event (i.e. MYC activation).^81,83^ Our current hypothesis is that the MYC establishes a background from which highly transformed and resistant cells emerge. The exact mechanism by which MYC creates this conducive cellular state remains unknown, but may involve long-term dysregulation of Ca^2+^ signaling,^62^ alterations in microRNA expression,^84^ or epigenetic reprogramming.^85^ Amplification of Ca^2+^ signalling through downregulation of Ca^2+^ transporters has been noted as a potential tumour promoting mechanism in other cancer types, including the Eμ-*myc* model of lymphoma.^62,63^

Overall, using our cell-based model, we were able to uncover novel targets within the fatty acid metabolism pathway that are prevalent in CL breast cancer and significantly correlate with poor patient survival. We predict that the uptake and oxidation of fatty acids is a crucial metabolic process for cell migration and a driving force behind the prevalence of a fatty acid metabolism gene signature in CL-TNBC. Future studies will examine the therapeutic potential of targeting fatty acid metabolism in CL-TNBC and how high-fat diets and obesity may influence fatty acid metabolism and tumour progression. There is also much to be learned concerning the cross talk between growth factor signalling, Ca^2+^ signalling, and metabolism. It is well known that many mitochondrial dehydrogenases and transporters contain Ca^2+^ binding sites and EF-hand motifs; however, the role of amplified Ca^2+^ signalling in mitochondrial function in cancer is largely unexplored.

## Supplementary information

Composite Supplementary Files

Supplementary Table S3

Supplementary Table S1

Supplementary Table S2

Supplementary Table S4

Supplementary Movie S1

Supplementary Movie S2

## Data Availability

Transcriptomic data will be deposited to the Gene Expression Omnibus (GEO) data repository. Other data that support the findings of this study are available from the corresponding author upon reasonable request.
