# Supplementary material for: MYC regulates fatty acid metabolism through a multigenic program in claudin-low triple negative breast cancer
Source: Br J Cancer. 2020 Jan 16;122(6):868–84. doi: 10.1038/s41416-019-0711-3 (PMC7078291; doi:10.1038/s41416-019-0711-3)
Supplement: Supplementary file 1 — Composite Supplementary Files [file 41416_2019_711_MOESM1_ESM.pdf]

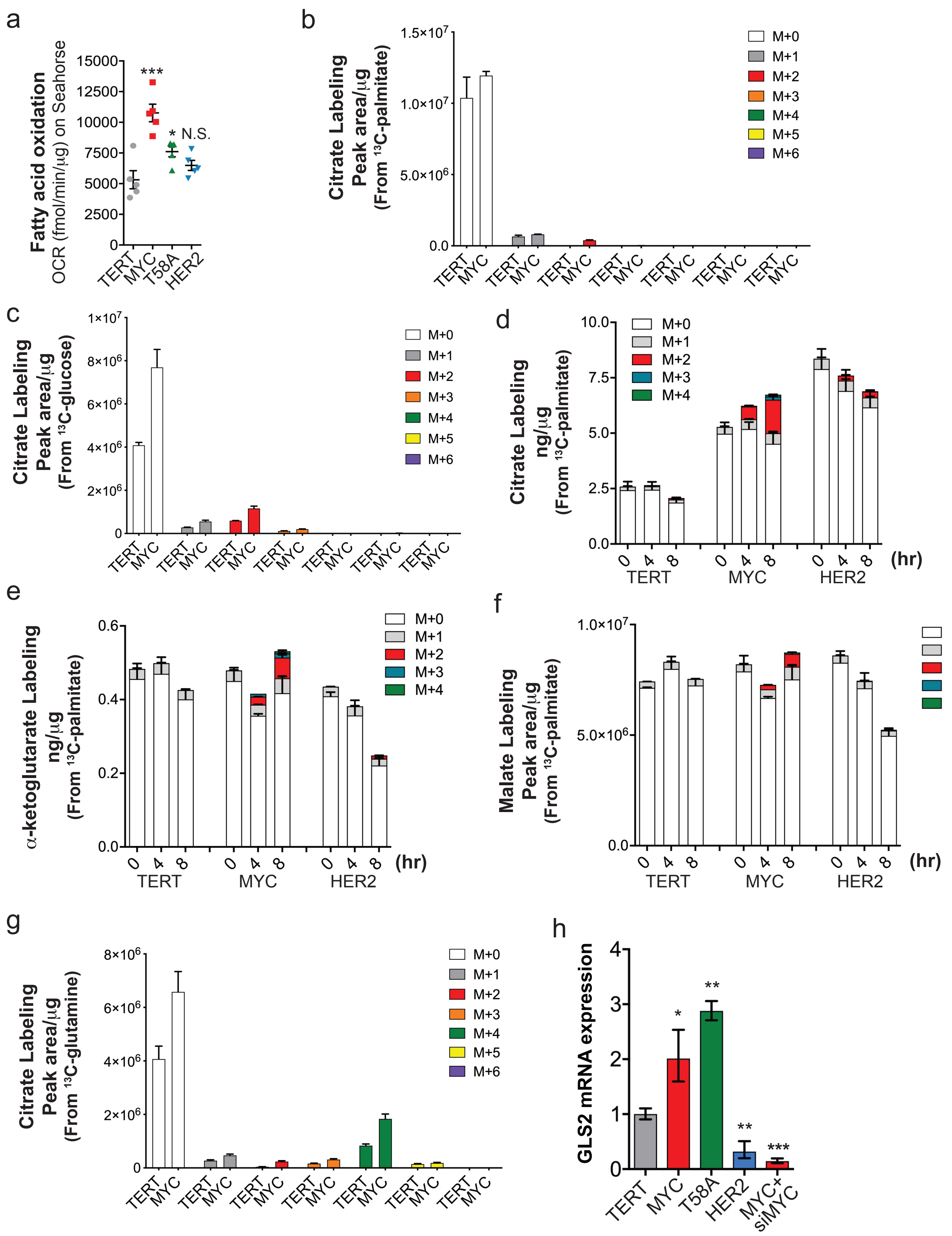
a
Fatty acid oxidation
OCR (fmol/min/μg) on Seahorse
15000
12500
10000
7500
5000
2500
0
***
*
N.S.
TERT
MYC
T58A
HER2
b
Citrate Labeling
Peak area/μg
(From 13C-palmitate)
1.5×10^7
1.0×10^7
5.0×10^6
0.0
M+0
M+1
M+2
M+3
M+4
M+5
M+6
TERT MYC
c
Citrate Labeling
Peak area/μg
(From 13C-glucose)
1×10^7
8×10^6
6×10^6
4×10^6
2×10^6
0
M+0
M+1
M+2
M+3
M+4
M+5
M+6
TERT MYC
d
Citrate Labeling
ng/μg
(From 13C-palmitate)
10.0
7.5
5.0
2.5
0.0
M+0
M+1
M+2
M+3
M+4
0 4 8 (hr)
TERT
MYC
HER2
e
α-ketoglutarate Labeling
ng/μg
(From 13C-palmitate)
0.6
0.4
0.2
0.0
M+0
M+1
M+2
M+3
M+4
0 4 8 (hr)
TERT
MYC
HER2
f
Malate Labeling
Peak area/μg
(From 13C-palmitate)
1.0×10^7
5.0×10^6
0.0
M+0
M+1
M+2
M+3
M+4
0 4 8 (hr)
TERT
MYC
HER2
g
Citrate Labeling
Peak area/μg
(From 13C-glutamine)
8×10^6
6×10^6
4×10^6
2×10^6
0
M+0
M+1
M+2
M+3
M+4
M+5
M+6
TERT MYC
h
GLS2 mRNA expression
4
3
2
1
0
*
**
**
***
TERT
MYC
T58A
HER2
MYC+ siMYC

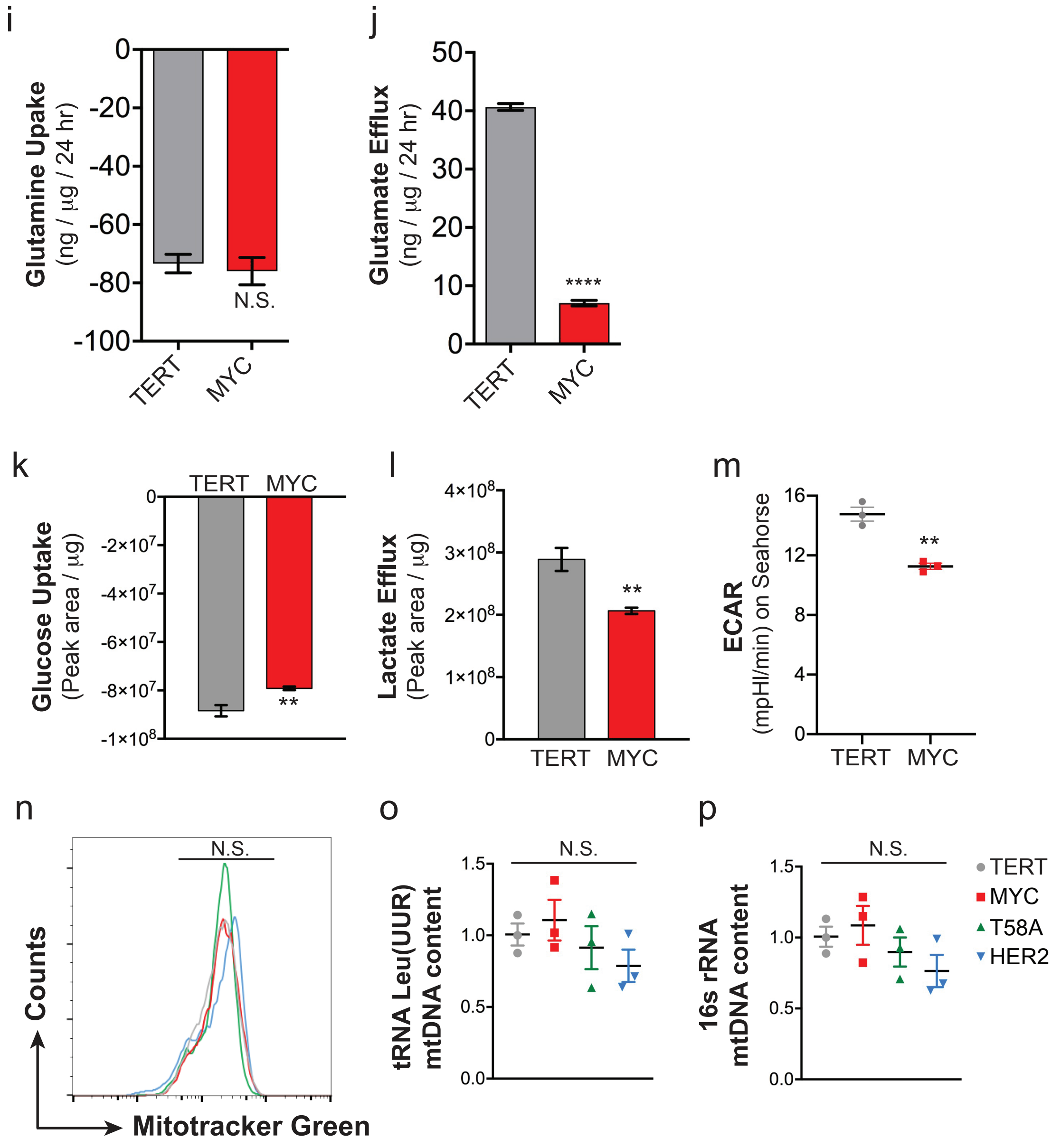
i
Glutamine Uptake
(ng / µg / 24 hr)
0
-20
-40
-60
-80
-100
N.S.
TERT
MYC
j
Glutamate Efflux
(ng / µg / 24 hr)
50
40
30
20
10
0
****
TERT
MYC
k
Glucose Uptake
(Peak area / µg)
TERT
MYC
0
-2×10^7
-4×10^7
-6×10^7
-8×10^7
-1×10^8
**
l
Lactate Efflux
(Peak area / µg)
4×10^8
3×10^8
2×10^8
1×10^8
0
**
TERT
MYC
m
ECAR
(mpHI/min) on Seahorse
16
12
8
4
0
**
TERT
MYC
n
N.S.
Counts
Mitotracker Green
o
N.S.
tRNA Leu(UUR)
mtDNA content
1.5
1.0
0.5
0.0
p
N.S.
16s rRNA
mtDNA content
1.5
1.0
0.5
0.0
TERT
MYC
T58A
HER2

Supplementary Figure S1. MYC HME cells engage in higher rates of FAO and glutamine oxidation, but do not have higher mitochondrial mass than TERT or HER2 HME cells. (**a**) Basal oxygen consumption rate (OCR) of HME cells. HME cells were cultured in the presence of 0.10 mM palmitate. n=5 independent experiments. Error bars represent mean ± standard error of the means (s.e.m.). p values were calculated using an ordinary one-way ANOVA with Tukey's multiple comparisons test. (**b-c**) LC-MS based quantification of citrate labeling from cells incubated with 0.10 mM U-13C-palmitate (b) or 5.5 mM U-13C-glucose (c) for 30 minutes. The carbon-13 labeling of citrate is presented as peak area for each isotopolog per μg of protein. n=3 independent samples that were measured in a single run on the mass spectrometer. (**d-f**) LC-MS based quantification of citrate labeling (d), α-ketoglutarate labeling (e) and malate labeling (f) in TERT, MYC and HER2 HME cells incubated with 0.05 mM U-13C-palmitate for 4 hours and 8 hours. The carbon-13 labeling is presented as a mass (ng) that was calculated relative to an external six point standard curve and is relative to protein content. n=3 independent samples that were measured in a single run on the mass spectrometer. (**g**) LC-MS based quantification of citrate labeling from cells incubated with 0.65 mM U-13C-glutamine for 30 minutes. The carbon-13 labeling of citrate is presented as peak area for each isotopolog and normalized to protein content. n=3 independent samples. Samples were measured in a single run on the mass spectrometer. (**h**) qRT-PCR analysis of mRNA expression of glutaminase 2 (GLS2) in HME cell lines. n=3 independent experiments. All values are relative to β-actin and normalized to TERT HME cells. Bars represent mean with upper and lower limits. p values were calculated using an unpaired student's t test. (**i-j**) LC-MS based quantification of the uptake of glutamine (i) and efflux of glutamate (j) in HME cells after 24 hours in culture. n=4 independent experiments. Samples were measured during a single run on the mass spectrometer and mass (ng) was calculated relative to an external six point standard curve. Error bars represent mean ± s.d. p values were calculated using an unpaired student's t test. (**k-l**) LC-MS based quantification of the uptake of glucose (k) and efflux of lactate (l) in HME cells after 24 hours in culture. n=4 independent experiments. Samples were measured during a single run on the mass spectrometer. Error bars represent mean ± s.d. p values were calculated using an unpaired student's t-test. (**m**) Extracellular acidification rate (ECAR) of HME cell lines. HME cells were cultured in Seahorse medium supplemented with 5.5 mM glucose and 0.65 mM glutamine, and 0.1 mM pyruvate in the absence of lipids (serum free). n=1 independent experiment performed in triplicate. Error bars represent mean ± s.e.m. p values were calculated using an unpaired student's t test. (**n**) Flow cytometric analysis of mitochondrial mass by Mitotracker Green FM dependent labeling of HME cells. Histogram represents one of n=3 independent experiments. (**o-p**) Mitochondrial DNA (mtDNA) content in HME cells. n=3 independent experiments. Error bars represent mean ± s.e.m. p values were calculated using an ordinary one-way ANOVA with Tukey's multiple comparisons test. * = $p < 0.05$, ** = $p < 0.01$, *** = $p < 0.001$, **** = $p < 0.0001$, N.S. = not significant.

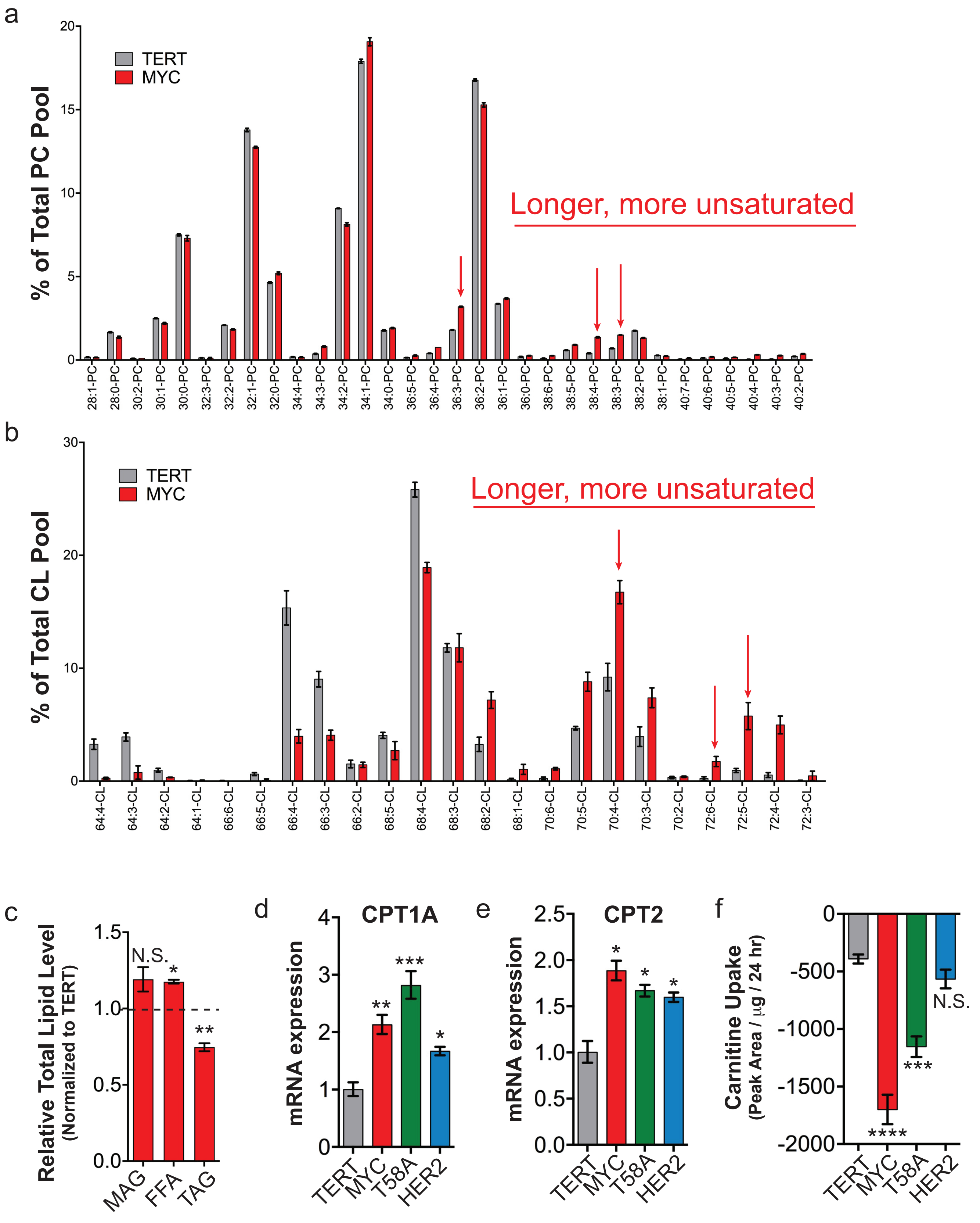
a
% of Total PC Pool
TERT
MYC
Longer, more unsaturated
0
5
10
15
20
28:1-PC
28:0-PC
30:2-PC
30:1-PC
30:0-PC
32:3-PC
32:2-PC
32:1-PC
32:0-PC
34:4-PC
34:3-PC
34:2-PC
34:1-PC
34:0-PC
36:5-PC
36:4-PC
36:3-PC
36:2-PC
36:1-PC
36:0-PC
38:6-PC
38:5-PC
38:4-PC
38:3-PC
38:2-PC
38:1-PC
40:7-PC
40:6-PC
40:5-PC
40:4-PC
40:3-PC
40:2-PC
b
% of Total CL Pool
TERT
MYC
Longer, more unsaturated
0
10
20
30
64:4-CL
64:3-CL
64:2-CL
64:1-CL
66:6-CL
66:5-CL
66:4-CL
66:3-CL
66:2-CL
68:5-CL
68:4-CL
68:3-CL
68:2-CL
68:1-CL
70:6-CL
70:5-CL
70:4-CL
70:3-CL
70:2-CL
72:6-CL
72:5-CL
72:4-CL
72:3-CL
c
Relative Total Lipid Level
(Normalized to TERT)
0.0
0.5
1.0
1.5
N.S.
*
**
MAG
FFA
TAG
d
CPT1A
mRNA expression
0
1
2
3
4
**
***
*
TERT
MYC
T58A
HER2
e
CPT2
mRNA expression
0.0
0.5
1.0
1.5
2.0
2.5
*
*
*
TERT
MYC
T58A
HER2
f
Carnitine Uptake
(Peak Area / μg / 24 hr)
0
-500
-1000
-1500
-2000
N.S.
***
****
TERT
MYC
T58A
HER2

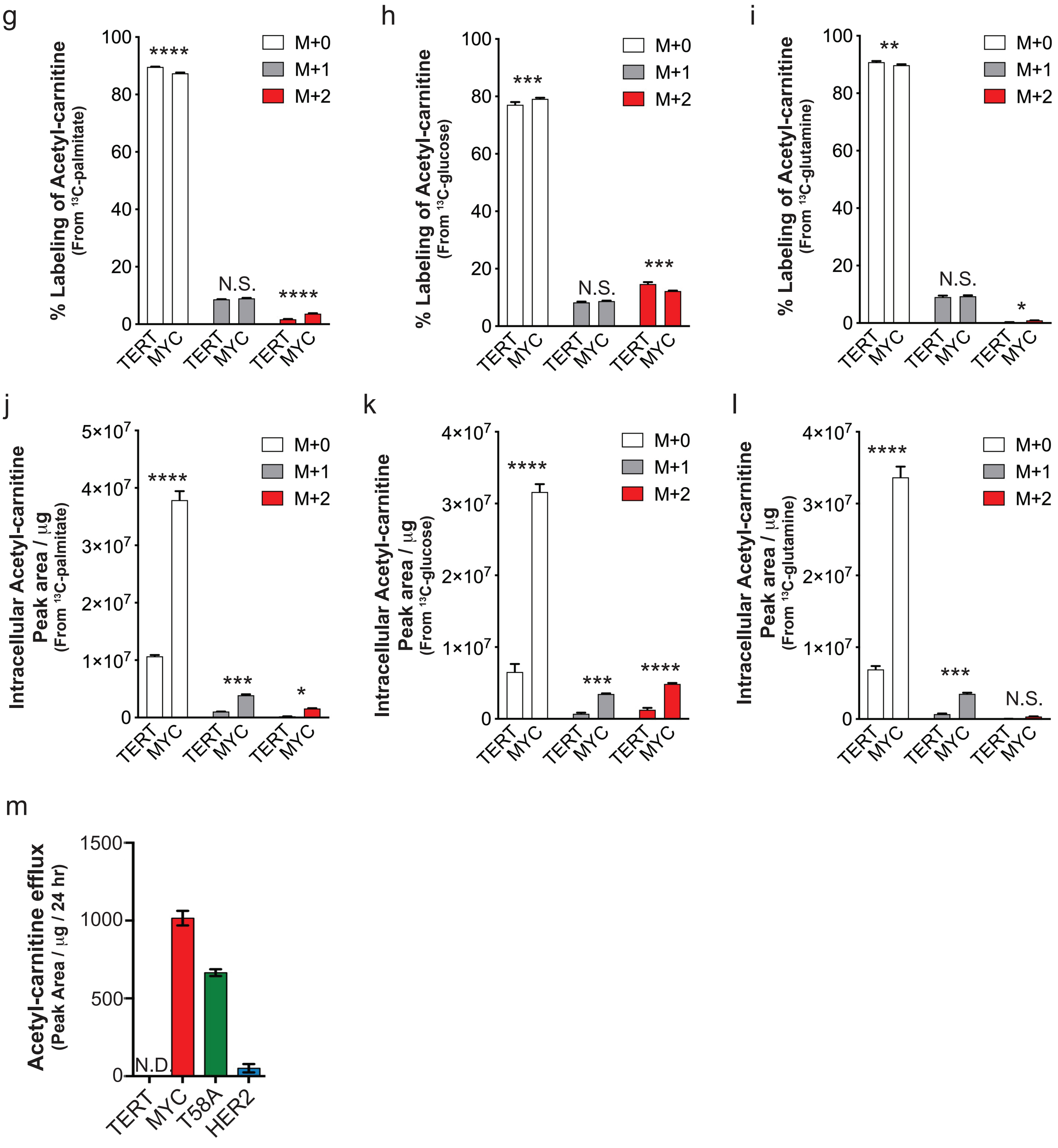
g
% Labeling of Acetyl-carnitine
(From 13C-palmitate)
100
80
60
40
20
0
M+0
M+1
M+2
****
N.S.
****
TERT
MYC
TERT
MYC
TERT
MYC
h
% Labeling of Acetyl-carnitine
(From 13C-glucose)
***
N.S.
***
i
% Labeling of Acetyl-carnitine
(From 13C-glutamine)
**
N.S.
*
j
Intracellular Acetyl-carnitine
Peak area / μg
(From 13C-palmitate)
5×10^7
4×10^7
3×10^7
2×10^7
1×10^7
****
***
*
k
Intracellular Acetyl-carnitine
Peak area / μg
(From 13C-glucose)
4×10^7
3×10^7
2×10^7
1×10^7
****
***
****
l
Intracellular Acetyl-carnitine
Peak area / μg
(From 13C-glutamine)
****
***
N.S.
m
Acetyl-carnitine efflux
(Peak Area / μg / 24 hr)
1500
1000
500
0
N.D.
TERT
MYC
T58A
HER2

Supplementary Figure S2. Lipid profiles of MYC HME cells indicate active scavenging and utilization of exogenous fatty acids for phospholipid synthesis. (**a-b**) Percent distribution of cardiolipin species (a) and phosphatidylcholine species (b) in TERT and MYC HME cells as quantified by LC-MS/MS based lipidomics. Cardiolipin molecules in MYC HME cells have longer fatty acyl chains that are also more unsaturated than TERT cells (see red arrows). n=3 independent experiments. Samples were measured during a single run on the mass spectrometer. Error bars represent mean ± s.d. (**c**) LC-MS based lipidomic profiling of TERT and MYC HME cells showing relative amounts of monoacylglycerol (MAG), free fatty acid (FFA), and triacylglycerol (TAG) in MYC HME cells. Data is normalized to TERT cells and relative to protein content. n=3 independent experiments. Error bars represent mean ± s.d. p values are calculated using a student's t test. (**d-e**) Relative mRNA expression of CPT1A (d) and CPT2 (e) in HME cells. Data are normalized to TERT and relative β-actin. n=3 independent experiments. Error bars represent mean with upper and lower limits. p values are calculated using a student's t test. (**f**) LC-MS based quantification of carnitine uptake from the media over 24 hours. Upward bars represent efflux and downward bars represent uptake. n=4 independent experiments. Error bars represent peak area mean ± s.d. Samples were measured in a single run on the mass spectrometer. p values were calculated using an ordinary one-way ANOVA with Tukey's multiple comparisons test. (**g-i**) LC-MS based quantification of acetyl-carnitine labeling from TERT and MYC HME cells treated with 0.05 mM U-13C-palmitate (g), 5.5 mM U-13C-glucose (h), and 0.65 mM U-13C-glutamine (i) for 30 minutes. The carbon-13 labeling is presented as a percent abundance of each isotopolog relative to the total acetyl-carnitine pool. n=3 independent samples. Samples were measured in a single run on the mass spectrometer. Adjusted p values were calculated using an ordinary two-way ANOVA and Sidak's multiple comparison test for each isotoplog. (**j-l**) Same data as in panels g, h and i, except the data is graphed as peak area per μg of protein instead of percent labeling. (**m**) LC-MS based quantification of acetyl-carnitine efflux into the media over 24 hours. Upward bars represent efflux and downward bars represent uptake. n=4 independent experiments. Error bars represent mean peak area ± s.d. Samples were measured in a single run on the mass spectrometer. * = $p<0.05$, ** = $p<0.01$, *** = $p<0.001$, **** = $p<0.0001$, N.S. = not significant, N.D. = not detected.

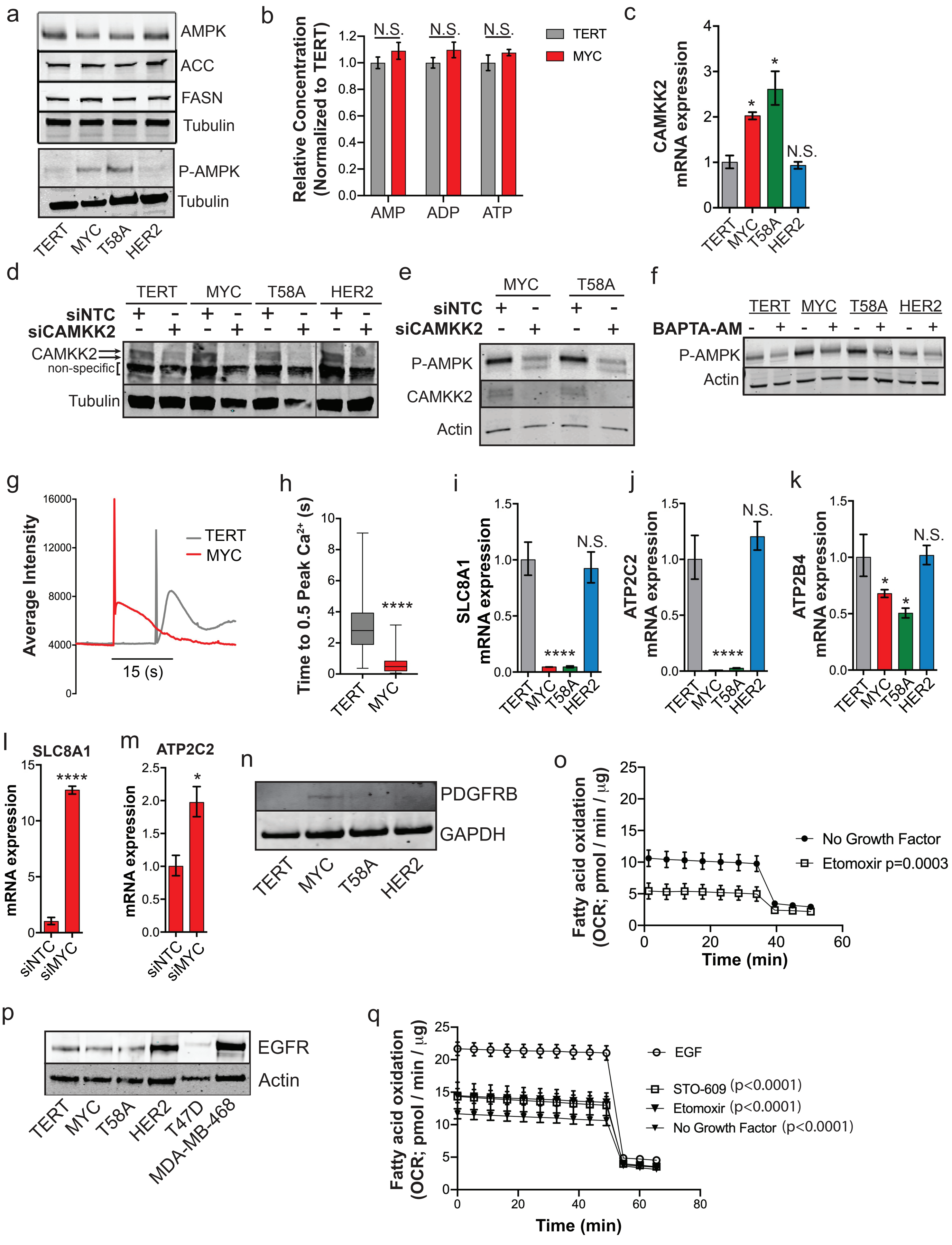
a
AMPK
ACC
FASN
Tubulin
P-AMPK
Tubulin
TERT
MYC
T58A
HER2
b
Relative Concentration (Normalized to TERT)
N.S.
N.S.
N.S.
1.2
1.0
0.8
0.6
0.4
0.2
0.0
AMP
ADP
ATP
TERT
MYC
c
CAMKK2 mRNA expression
4
3
2
1
0
*
*
N.S.
TERT
MYC
T58A
HER2
d
TERT
MYC
T58A
HER2
siNTC
siCAMKK2
CAMKK2
non-specific
Tubulin
e
MYC
T58A
siNTC
siCAMKK2
P-AMPK
CAMKK2
Actin
f
TERT
MYC
T58A
HER2
BAPTA-AM
P-AMPK
Actin
g
Average Intensity
16000
12000
8000
4000
0
TERT
MYC
15 (s)
h
Time to 0.5 Peak Ca2+ (s)
10
8
6
4
2
0
****
TERT
MYC
i
SLC8A1 mRNA expression
1.5
1.0
0.5
0.0
****
N.S.
TERT
MYC
T58A
HER2
j
ATP2C2 mRNA expression
1.5
1.0
0.5
0.0
****
N.S.
TERT
MYC
T58A
HER2
k
ATP2B4 mRNA expression
1.5
1.0
0.5
0.0
*
*
N.S.
TERT
MYC
T58A
HER2
l
SLC8A1
mRNA expression
15
10
5
0
****
siNTC
siMYC
m
ATP2C2
mRNA expression
2.5
2.0
1.5
1.0
0.5
0.0
*
siNTC
siMYC
n
PDGFRB
GAPDH
TERT
MYC
T58A
HER2
o
Fatty acid oxidation (OCR; pmol / min / µg)
25
20
15
10
5
0
0
20
40
60
Time (min)
No Growth Factor
Etomoxir p=0.0003
p
EGFR
Actin
TERT
MYC
T58A
HER2
T47D
MDA-MB-468
q
Fatty acid oxidation (OCR; pmol / min / µg)
25
20
15
10
5
0
0
20
40
60
80
Time (min)
EGF
STO-609 (p<0.0001)
Etomoxir (p<0.0001)
No Growth Factor (p<0.0001)

Supplementary Figure S3. Ca^{2+} dependent activation of CAMKK2 stimulates fatty acid oxidation in MYC HME cells. (**a**) Immunoblot analysis of AMPK, ACC, FASN and phosphorylated AMPK (P-AMPK) in HME cells. Tubulin was used as a loading control. (**b**) LC-MS based quantification of intracellular AMP, ADP and ATP levels in TERT and MYC HME cells. n=3 independent experiments. Samples were measured during a single run on the mass spectrometer. Error bars represent mean ± s.d. p values were calculated using an unpaired student's t-test. (**c**) Relative mRNA expression of CAMKK2 in HME cells. Data is normalized to TERT HME cells and relative to β-actin. n=3 independent experiments. Error bars represent mean with upper and lower limits. p values were calculated using an unpaired student's t-test. (**d**) CAMKK2 protein expression in HME cells. Non-specific bands were determined by siRNA-mediated knockdown of CAMKK2 expression in each HME cell line. (**e**) CAMKK2 and P-AMPK protein expression in MYC and T58A HME cells transfected with a non-targeting control siRNA (siNTC) or a pool of four individual siRNAs against CAMKK2 (siCAMKK2). (**f**) Expression of P-AMPK in HME cells after chelation of intracellular Ca2+ by 20 μM BAPTA-AM. (**g**) Representative single cell traces of the intracellular [Ca^{2+}] changes in response to release of caged-IP_3 by a UV pulse. (**h**) Box and whisker plot showing the time from caged-IP_3 release to half maximal peak Ca^{2+} response. Boxes represent 25th to 75th percentile, whiskers represent min to max, and lines represent medians. p values were calculated using an unpaired student's t-test. (**i-k**) Relative mRNA expression of SLC8A1, ATP2C2, and ATP2B4 in HME cells normalized to TERT HME cells and relative to β-actin. n=3 independent experiments. Error bars represent mean with upper and lower limits. p values were calculated using an unpaired student's t-test. (**l-m**) Relative mRNA expression of SLC8A1 (l) and ATP2C2 (m) in MYC HME cells transfected with a non-targeting control siRNA (siNTC) or a pool of four individual siRNAs against MYC (siMYC) normalized siNTC and relative to β-actin. n=3 independent experiments. Error bars represent mean with upper and lower limits. p values were calculated using an unpaired student's t-test. (**n**) Immunoblot analysis of PDGFRB protein expression in HME cells. (**o**) Basal OCR of MYC HME cells in the absence of growth factor +/- 40 μM etomoxir. (**p**) Immunoblot analysis of EGFR protein expression in HME cells. T47D and MDA-MD-468 cell lines were used as a negative and positive control for EGFR expression, respectively. (**q**) Basal OCR of HME cells treated with 25 ng/ml EGF and treated with vehicle, STO-609 (10 μM) or etomoxir (40 μM). All OCR measurements were done in the presence of 0.10 mM palmitate. n=5 replicates. Error bars represent mean ± s.e.m. Total oxygen consumption was calculated based on the area under the curve for each sample. The average of total oxygen consumption was compared between groups using ANOVA with post-hoc multiple comparisons. Unadjusted p values are reported. * = $p<0.05$, ** = $p<0.01$, *** = $p<0.001$, **** = $p<0.0001$, N.S. = not significant.

a

TERT	SubG1	G0/G1	S	G2/M
Untreated	0.37	62.90	8.04	27.78
STO-609	0.22	45.60	2.49	51.34
Etomoxir	1.04	68.66	1.80	28.08

MYC	SubG1	G0/G1	S	G2/M
Untreated	2.87	57.11	16.37	23.64
STO-609	23.18	41.22	12.54	22.66
Etomoxir	46.00	50.88	1.72	1.47

T58A	SubG1	G0/G1	S	G2/M
Untreated	1.29	56.47	15.60	25.46
STO-609	14.90	53.84	7.99	22.69
Etomoxir	32.01	44.81	12.12	10.83

HER2	SubG1	G0/G1	S	G2/M
Untreated	3.33	60.73	16.04	19.60
STO-609	8.48	53.48	11.22	26.31
Etomoxir	11.31	75.52	3.58	10.12

b

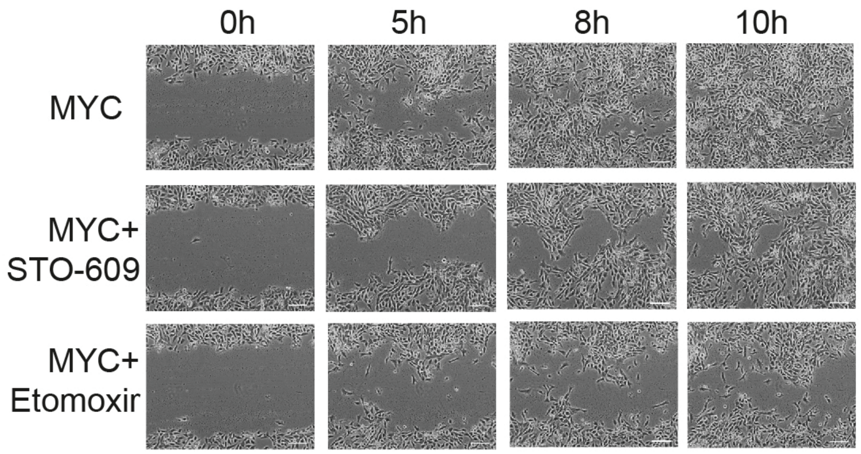

c

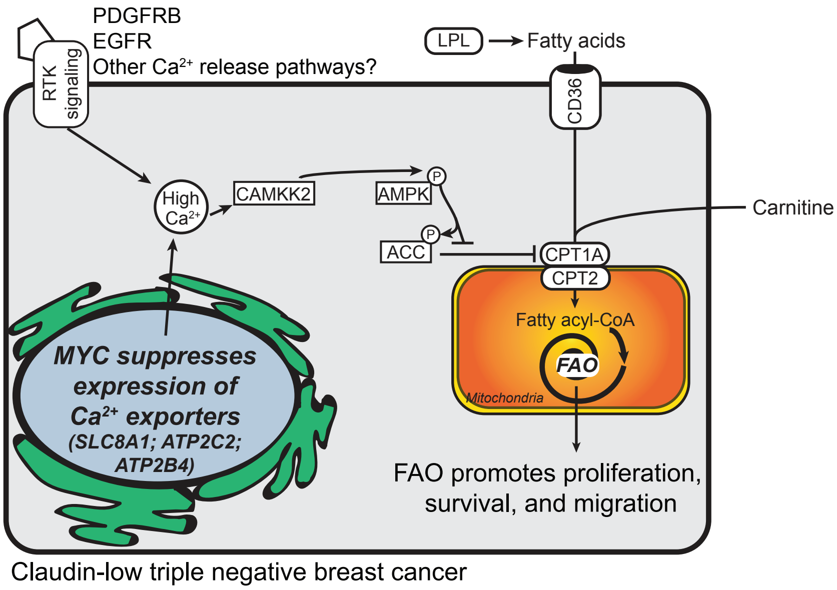

Supplementary Figure S4. Inhibition of CAMKK2 causes cytostatic and cytoxic effects and impacts cell migration. (**a**) Quantification of cell cycle distribution data from Figure 4a-d. (**b**) Scratch assay on MYC HME cells that were either untreated or treated with 10 μM STO-609 or 125 μM etomoxir for up to 10 hours. (**c**) Graphical illustration highlighting the signaling and metabolic pathways impacted by changes in fatty acid metabolism and Ca2+ signaling in MYC HME cells. RTK induced Ca2+ release activates CAMKK2 which then initiates a signaling cascade that promotes FAO. In addition, high expression of CD36, LPL and other fatty acid metabolism genes facilitates the uptake and oxidation of fatty acids. FAO promotes proliferation, survival, and migration.

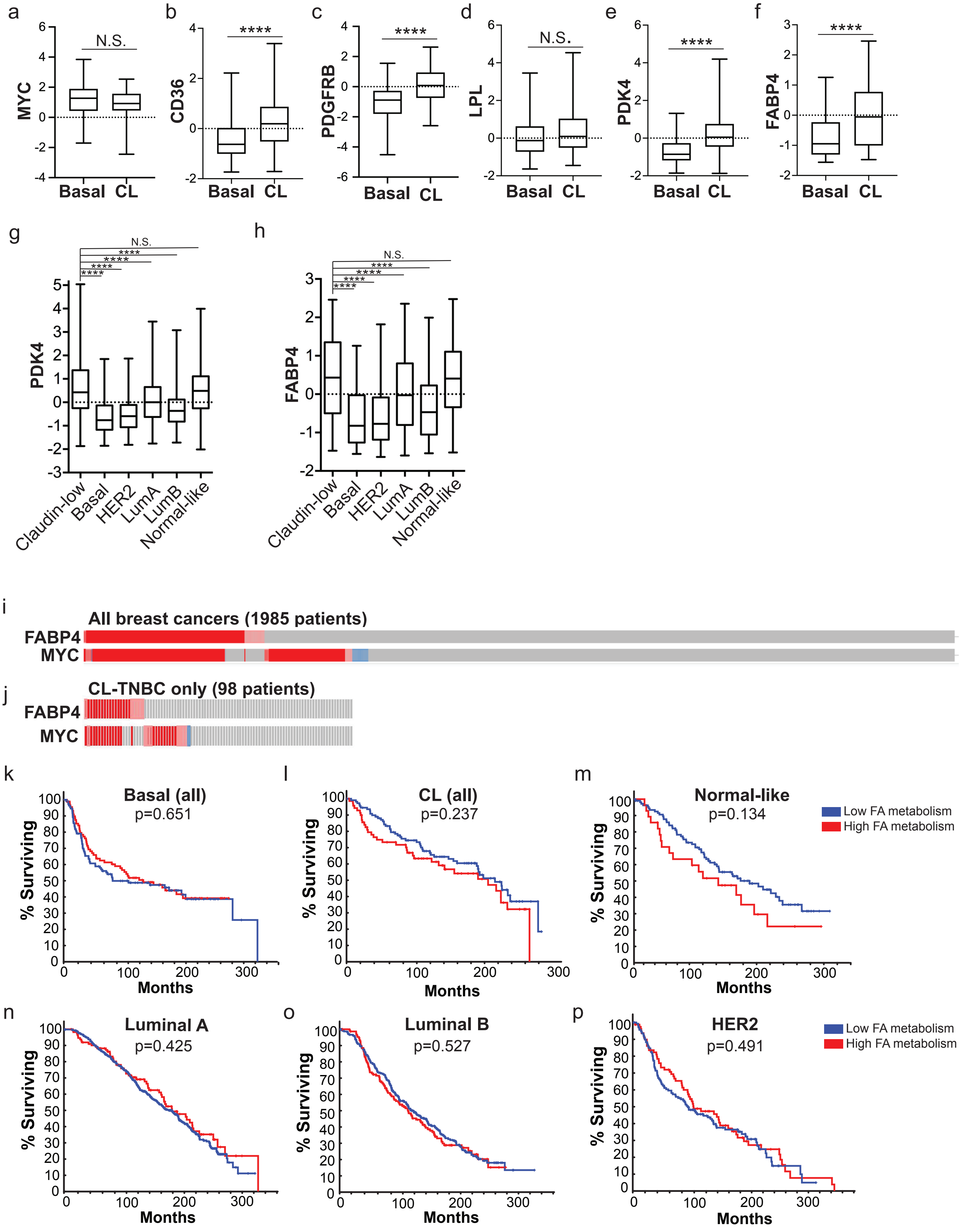

a
MYC
N.S.
Basal CL
b
CD36
****
Basal CL
c
PDGFRB
****
Basal CL
d
LPL
N.S.
Basal CL
e
PDK4
****
Basal CL
f
FABP4
****
Basal CL
g
PDK4
N.S.
****
Claudin-low
Basal
HER2
LumA
LumB
Normal-like
h
FABP4
N.S.
****
Claudin-low
Basal
HER2
LumA
LumB
Normal-like
i
All breast cancers (1985 patients)
FABP4
MYC
j
CL-TNBC only (98 patients)
FABP4
MYC
k
Basal (all)
p=0.651
% Surviving
Months
l
CL (all)
p=0.237
% Surviving
Months
m
Normal-like
p=0.134
% Surviving
Months
Low FA metabolism
High FA metabolism
n
Luminal A
p=0.425
% Surviving
Months
o
Luminal B
p=0.527
% Surviving
Months
p
HER2
p=0.491
% Surviving
Months
Low FA metabolism
High FA metabolism

Supplementary Figure S5. High expression of fatty acid metabolism genes predicts poor survival in CL-TNBC patients. (**a-f**) Box and whisker plots of mRNA expression (z-scores) for MYC (a), CD36 (b), PDGFRB (c), LPL (d), PDK4 (e), and FABP4 (f) in basal-TNBC compared to CL-TNBC patient samples. Expression data was extracted from the METABRIC database. Boxes represent 25th to 75th percentile, whiskers represent min to max, and lines represent medians. Basal-TNBC, n=151. CL-TNBC, n=99. p values were calculated using an unpaired student's t-test. (**g-h**) Box and whisker plots of PDK4 (g) and FABP4 (h) mRNA expression levels (z-scores) in human breast cancer patient tumors extracted from the METABRIC database. Boxes represent 25th to 75th percentile, whiskers represent min to max, and lines represent medians. p values were determined using a Dunnett's multiple comparison test. Tumors were classified based on the PAM50+claudin-low molecular subtyping of breast cancer as called by the METABRIC database. Claudin-low, n=182; Basal, n=198; HER2, n=218; Luminal A, n=673; Luminal B, n=454; Normal-like, n=135. (**i-j**) Oncoprints of FABP4 and MYC copy number alterations and mRNA expression in all breast cancers (i) and CL-TNBC (j). Dark red bars indicate copy number gains and gene amplification. Light red bars indicate upregulation. Light blue bars indicate downregulation. Dark blue bars indicate homozygous deletion. Gray bars indicates no change. FABP4 and MYC are commonly co-amplified in breast cancer. (**k-p**) Kaplan-Meier survival plots of breast cancer patients. Patients were stratified into either low expression of fatty acid metabolism genes or high expression of fatty acid metabolism genes based on the expression and copy number alterations of CD36, LPL, PDK4, and FABP4. p values were calculated using log-rank test. * = p<0.05, ** = p<0.01, *** = p<0.001, **** = p<0.0001, N.S. = not significant.

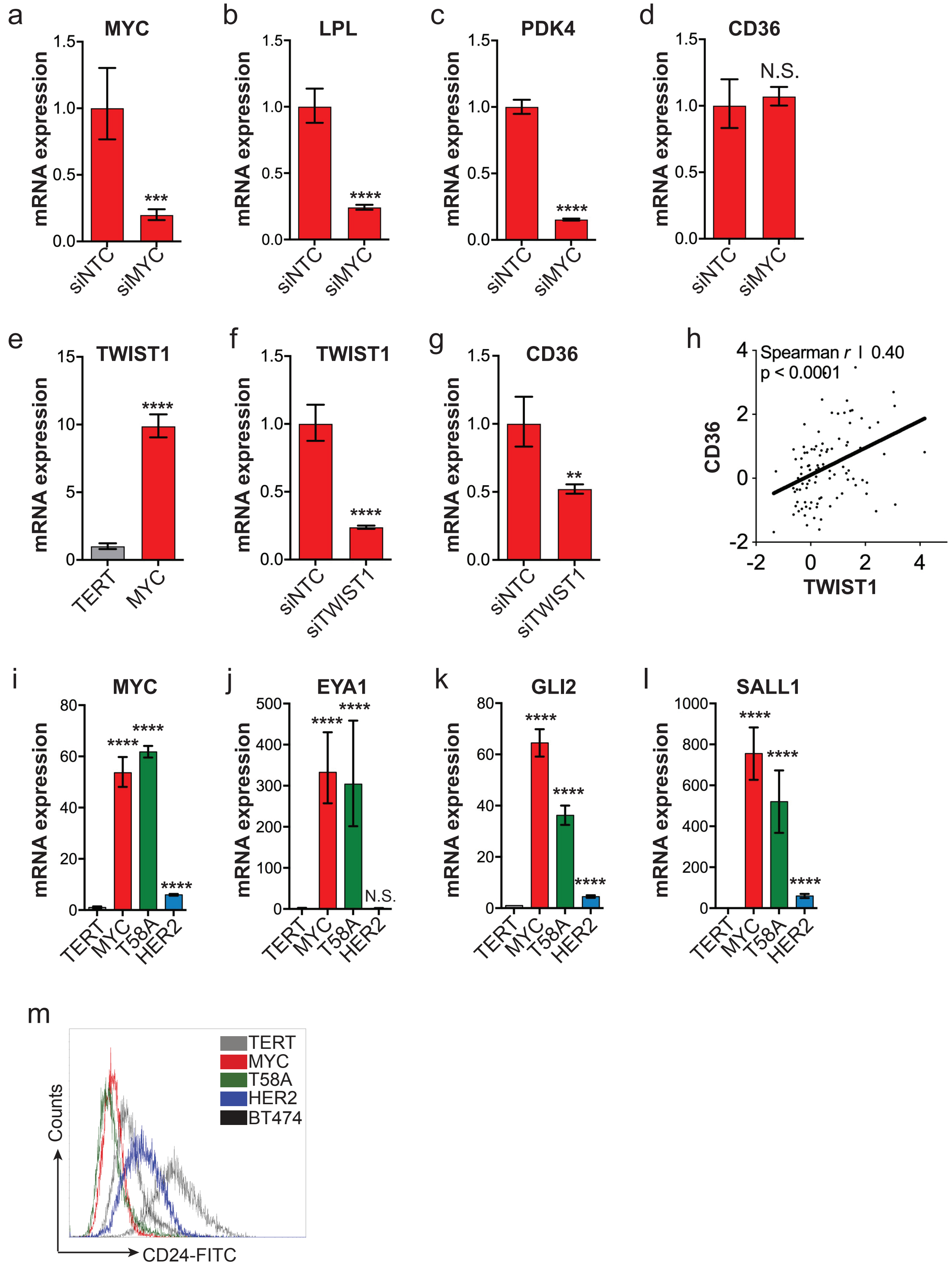
a
MYC
mRNA expression
siNTC
siMYC
***
b
LPL
****
c
PDK4
****
d
CD36
N.S.
e
TWIST1
TERT
MYC
****
f
TWIST1
siNTC
siTWIST1
****
g
CD36
**
h
Spearman r = 0.40
p < 0.0001
CD36
TWIST1
i
MYC
TERT
MYC
T58A
HER2
****
j
EYA1
N.S.
k
GLI2
l
SALL1
m
TERT
MYC
T58A
HER2
BT474
Counts
CD24-FITC

Supplementary Figure S6. High expression of fatty acid metabolism genes predicts poor survival in CL-TNBC patients. (**a-d**) Relative mRNA expression of MYC (a), LPL (b), PDK4 (c), and CD36 (d) in MYC HME cells transfected with a non-targeting control siRNA (siNTC) or a pool of four individual siRNAs against MYC (siMYC) normalized siNTC and relative to β-actin. n=3 independent experiments. Error bars represent mean with upper and lower limits. p values were calculated using an unpaired student's t-test. (**e**) Relative mRNA expression of TWIST1 in TERT and MYC HME cells normalized to TERT HME cells and relative to β-actin. n=3 independent experiments. Error bars represent mean with upper and lower limits. p values were calculated using an unpaired student's t-test. (**f-g**) Relative mRNA expression of TWIST (f) and CD36 (g) in MYC HME cells transfected with a non-targeting control siRNA (siNTC) or a pool of four individual siRNAs against TWIST1 (siTWIST1) normalized to siNTC and relative to β-actin. n=3 independent experiments. Error bars represent mean with upper and lower limits. p values were calculated using an unpaired student's t-test. (**h**) Scatter plots of mRNA expression of CD36 against TWIST1 in CL-TNBC (n = 99). Black line represents linear regression of the scatter plot. The Spearman correlation r value and p value are reported on the graph. (**i-l**) Relative mRNA expression of MYC (i), EYA1 (j), GLI2 (k), and SALL1 (l) in HME cells normalized to TERT HME cells and relative to β-actin. n=3 independent experiments. Error bars represent mean with upper and lower limits. p values were calculated using an unpaired student's t-test. (**m**) Surface expression of breast cancer stemness marker CD24 on HME cells. BT474, an ER, PR, and HER2 triple positive cell line, was used as a positive control. CD24 is commonly downregulated in CL-TNBC . *p<0.05, **p<0.01, ***p<0.001, ****p<0.0001

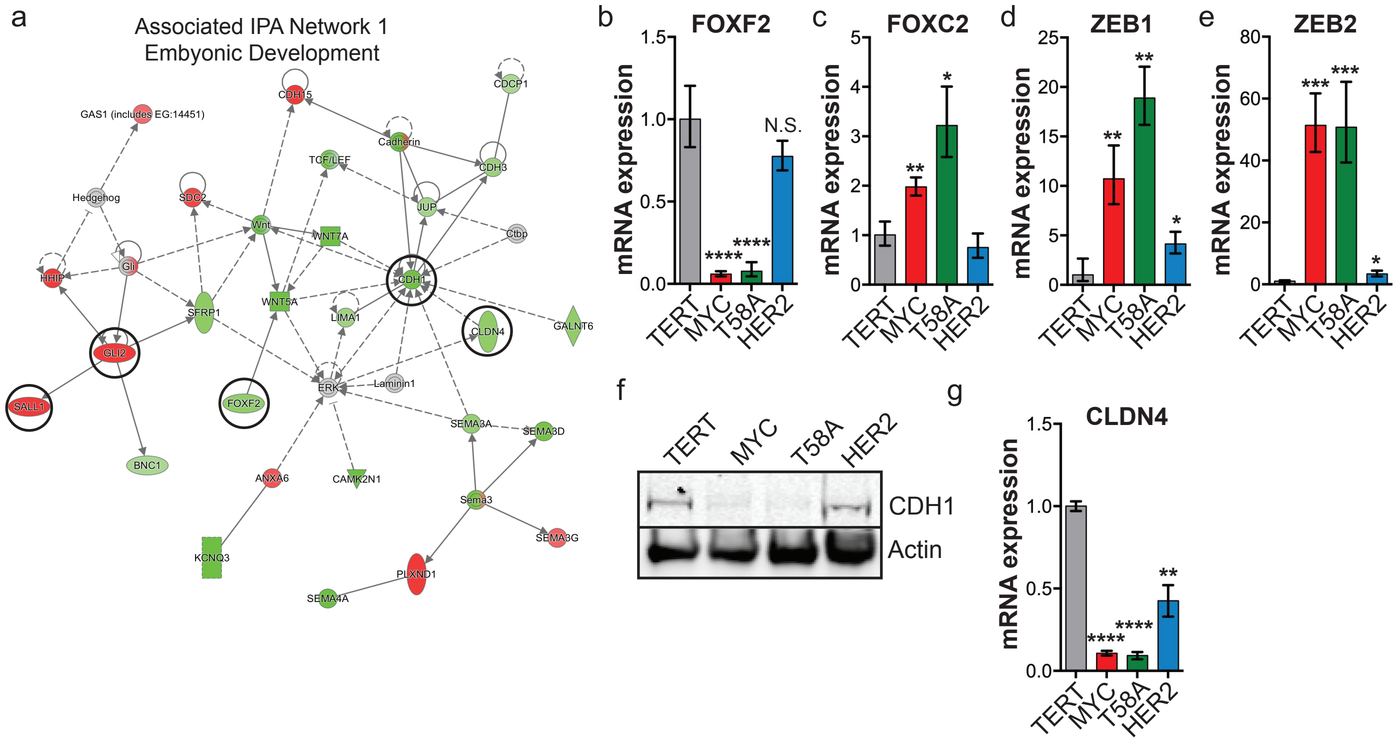

Supplementary Figure S7. MYC HME cells express epithelia-to-mesenchymal markers and have low expression of cell adhesion proteins. (**a**) Interaction map of the organismal and embryonic developmental gene network in MYC HME cells (IPA Network 1 from Supplementary Table S3). Ingenuity Pathway Analysis was used to create the interaction map. Black circles highlight genes involved in hedgehog signaling, EMT, and cell adhesion. (**b-e**) Relative mRNA expression of FOXF2 (b), FOXC2 (c), ZEB1 (d), and ZEB2 (e) in HME cells normalized to TERT and relative to β-actin. n=3 independent experiments. Error bars represent mean with upper and lower limits. p values were calculated using an unpaired student's t-test. (**f**) Immunoblot analysis of E-cadherin (CHD1) protein expression in HME cell lines. (**g**) Relative mRNA expression of claudin-4 (CLDN4) in HME cells. Data is normalized TERT and relative to β-actin. n=3 independent experiments. Error bars represent mean with upper and lower limits. p values were calculated using an unpaired student's t-test. * = $p<0.05$, ** = $p<0.01$, *** = $p<0.001$, **** = $p<0.0001$, N.S. = not significant.

a

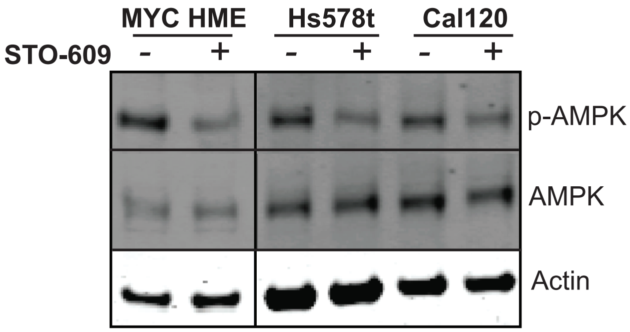

Supplementary Figure S8. STO-609 inhibits phosphorylation of AMPK in claudin-low triple negative breast cancer cells. (a) Immunoblot for expression of P-AMPK and total AMPK in MYC HME cells, and two different CL-TNBC cell lines, Hs578t and Cal120. Cells were treated for two hours with the CAMKK2 inhibitor STO-609 (10 μM). All samples are from the same exposure on the same immunoblot.

SUPPLEMENTARY TABLE LEGENDS

Supplementary Table S1. RNA sequencing data showing normalized expression values for genes in TERT and MYC HME cells.

Supplementary Table S2. RNA sequencing data showing normalized expression values for genes in TERT and HER2 cells.

Supplementary Table S3. Ingenuity pathway analysis network scores for the top five gene networks in TERT versus MYC HME cells and TERT versus HER2 cells. Data are based on RNA sequencing data in Supplementary Tables S1 and S2 and created by Ingenuity Pathway Analysis.

Supplementary Table S4. 163 lipid metabolism gene list with fold change relative to TERT HME cells for MYC HME cells and HER2 HME cells. This table is in reference to the data in Figure 2c.

SUPPLEMENTARY MOVIE LEGENDS

Supplementary Movie S1. TERT HME cells were monitored for 6 hours following addition of BODIPY-C12.

Supplementary Movie S2. MYC HME cells were monitored for 6 hours following addition of BODIPY-C12. Note the higher fluorescence, particularly during cell division, and the much higher rate of migration for MYC HME cells compared to TERT HME cells in Supplementary Movie S1.
